# Genetic Diversity of *Giardia duodenalis*: Multilocus Genotyping Reveals Zoonotic Potential between Clinical and Environmental Sources in a Metropolitan Region of Brazil

**DOI:** 10.1371/journal.pone.0115489

**Published:** 2014-12-23

**Authors:** Mauricio Durigan, Aluana Gonçalves Abreu, Maria Imaculada Zucchi, Regina Maura Bueno Franco, Anete Pereira de Souza

**Affiliations:** 1 Molecular Biology and Genetic Engineering Center (CBMEG), University of Campinas (UNICAMP), Campinas, SP, Brazil; 2 APTA - São Paulo Agency for Agribusiness Technology, Piracicaba, SP, Brazil; 3 Department of Animal Biology, Biology Institute, University of Campinas (UNICAMP), Campinas, SP, Brazil; 4 Department of Plant Biology, Biology Institute, University of Campinas (UNICAMP), Campinas, SP, Brazil; Food and Drug Administration, United States of America

## Abstract

**Background:**

*Giardia duodenalis* is a flagellate protozoan that parasitizes humans and several other mammals. Protozoan contamination has been regularly documented at important environmental sites, although most of these studies were performed at the species level. There is a lack of studies that correlate environmental contamination and clinical infections in the same region. The aim of this study is to evaluate the genetic diversity of a set of clinical and environmental samples and to use the obtained data to characterize the genetic profile of the distribution of *G. duodenalis* and the potential for zoonotic transmission in a metropolitan region of Brazil.

**Methodology/Principal Findings:**

The genetic assemblages and subtypes of *G. duodenalis* isolates obtained from hospitals, a veterinary clinic, a day-care center and important environmental sites were determined via multilocus sequence-based genotyping using three unlinked gene loci. Cysts of *Giardia* were detected at all of the environmental sites. Mixed assemblages were detected in 25% of the total samples, and an elevated number of haplotypes was identified. The main haplotypes were shared among the groups, and new subtypes were identified at all loci. Ten multilocus genotypes were identified: 7 for assemblage A and 3 for assemblage B.

**Conclusions/Significance:**

There is persistent *G. duodenalis* contamination at important environmental sites in the city. The identified mixed assemblages likely represent mixed infections, suggesting high endemicity of *Giardia* in these hosts. Most *Giardia* isolates obtained in this study displayed zoonotic potential. The high degree of genetic diversity in the isolates obtained from both clinical and environmental samples suggests that multiple sources of infection are likely responsible for the detected contamination events. The finding that many multilocus genotypes (MLGs) and haplotypes are shared by different groups suggests that these sources of infection may be related and indicates that there is a notable risk of human infection caused by *Giardia* in this region.

## Introduction

*Giardia duodenalis* (syn. *G. lamblia* and *G. intestinalis*) is a flagellate unicellular protozoan that parasitizes both humans and several domestic and wild animal species. The *Giardia* genus consists of six species that have been characterized based on their morphology and trophozoite ultrastructure. The species identified to date are *G. agilis*, which parasitizes amphibians; *G. ardeae* and *G. psittaci*, which parasitize birds; *G. microti* and *G. muris*, which parasitize rodents; and *G. duodenalis*, which parasitizes mammals [1]–[3]. Giardiasis is one of the most common waterborne diseases in the world. The cysts are transmitted via the fecal-oral rote through contaminated water or food, by contact with infected people or animals or through sexual contact.

Approximately 1.2 million new cases of giardiasis occur annually in the United States alone [4], and 280 million people per year are estimated to present this infection globally [5]. The high prevalence of this infection in developed countries and the increasing frequency of outbreaks in day-care centers have led to the inclusion of giardiasis in the World Health Organization's (WHO) Neglected Disease Initiative [6], and *Giardia* is now considered a re-emerging infectious agent [7]. Over 1.7 million people die every year from poor hygiene, a lack of sanitary infrastructure and a lack of clean water or sewage treatment [8]. Diarrheal diseases, including giardiasis, account for 15% of all deaths in children younger than 5 years of age [9].

The available data on the prevalence of *G. duodenalis* in non-human hosts are primarily confined to dogs, cats and livestock. Although human infections are of primary interest, *Giardia* has been reported in dogs and cats worldwide [10]. Surveys addressing the presence of *Giardia* infections in dogs have reported a high prevalence [11], [12]; the prevalence in cats is also of great concern from both a clinical and public health perspective [13], [14]. Similarly, *Giardia* infection in livestock is common and may be an important cause of economic losses, because its prevalence can reach 100% in calves [15]–[17].

Outbreaks of human giardiasis are frequently caused by contamination of drinking water [18] and a high prevalence of *Giardia* has been detected in surface waters [19], [20]. However, few studies have assessed the levels of clinical or environmental contamination. Four densely urbanized regions of São Paulo State were found to present higher rates than are tolerated by the United States Environmental Protection Agency (USEPA) regarding the risk of infection. Within this scenario, the metropolitan region of Campinas exhibited the highest percentage of positivity for *Giardia* and the highest risk of infection among these regions [21]. The detection of *Giardia* in surface waters may provide important clues regarding the environmental epidemiology of the parasite [22], especially in developing countries, where water and sewage treatment rates remain low.

*G. duodenalis* shows a high degree of genetic diversity, and isolates of this species have been grouped into genetic assemblages [23]. Although studies investigating the genetic diversity of *Giardia* were initiated through allozymes [24] and restriction fragment length polymorphism (RFLP) analyses [25], the genetic assemblages of *G. duodenalis* are now mainly identified using PCR followed by sequencing of conserved genes such as glutamate dehydrogenase, triose phosphate isomerase, beta-giardin, elongation factor 1-alpha and small subunit ribosomal RNA (ssu-rRNA) [26]. Single nucleotide polymorphisms (SNPs) identified in amplified fragments of these genes have enabled the characterization of *Giardia* isolates in different genetic assemblages. *G. duodenalis* assemblages A and B show zoonotic potential and can parasitize many hosts, such as humans, dogs, cats, non-human primates, livestock, horses, pigs, deer, moose and ferrets. Cysts recovered from human samples have always been grouped into one of two major genetic assemblages (A and B) that have been described worldwide [24], [25], [27]–[30]. Molecular studies have revealed the existence of genetic structure within these major assemblages [31]. Approximately three-quarters of assemblage A sequences obtained from non-human hosts, such as livestock and companion animals, correspond to sub-assemblage AI. In humans, a similar proportion of assemblage A sequences have been genotyped as belonging to sub-assemblage AII [12], [32]. Assemblage B sub-assemblages BIII and BIV have been observed in cattle, horses, monkeys and dogs. In humans, the two sub-assemblages are observed at similar frequencies. Sub-assemblage AIII was first identified in wild hoofed stock and is restricted to animals. Thus, sub-assemblages AI, AII, BIII and BIV are potentially zoonotic [12], [32]–[34]. The other assemblages are commonly associated with specific hosts. For example, assemblages C and D have been identified in dogs, cats, coyotes and wolves; assemblage E has been found in livestock; assemblage F has been found in cats; and assemblage G has been found in rats [26], [31]. Recently, another assemblage (H) was identified in marine vertebrates as a sister clade to assemblage G based on an analysis of the *gdh* gene [35].

In the present study, *Giardia* spp. cysts obtained from multiple hosts and collected from different locations in urban areas of the metropolitan region of Campinas in the state of São Paulo, Brazil were genetically characterized based on multilocus analyses. The aim of this study is to determine the genetic diversity of a set of clinical and environmental samples and to use these data to assess the potential for zoonotic transmission and mixed infection and their possible impacts on public health.

## Methods

### Study area and design

**Figure 1:**
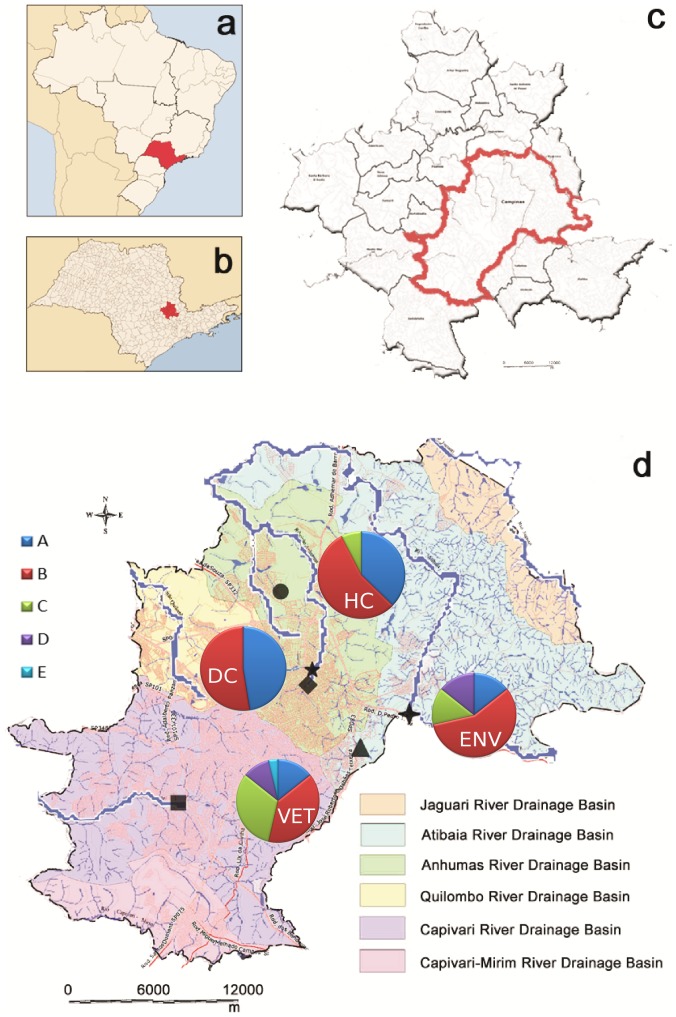
Collection sites of positive samples. Brazil (a), state of São Paulo (b), metropolitan region of Campinas (c) and the collection sites of positive samples plotted on a map of the city of Campinas colored to indicate the various drainage basins (d). The assemblages identified in each group were plotted in the map close to the collection site. •Unicamp Hospital and hospital sewage. ⧫Proença stream, Serafim stream and day-care center. ▪Ouro Verde Hospital. ⧫Campinas water abstraction site. ★Anhumas River. ▴Samambaia waste water treatment plant.

Samples from multiple sites and hosts were collected to genetically characterize the cysts of the protozoan parasite *Giardia* spp. The Campinas metropolitan region in the state of São Paulo in southeastern Brazil consists of 20 cities that are home to nearly 3 million people. Campinas is the main city and includes three primary urban drainage basins that drain the majority of the water in this region. Environmental samples were collected from correlated water sources including rivers, urban streams, hospital sewage, a waste water treatment plant and the water abstraction site for the city from the Atibaia River. Clinical samples were obtained through partnerships with hospitals, a day-care center, a veterinary clinic and a farm, all of which provided positive samples (Fig. 1). Together, these partnerships allowed us to survey a greater number of parasitized hosts, thereby increasing the diversity of the sampled *Giardia* cysts. Because our collaborators provided positive samples, the focus of this study was not to present information about the prevalence of *Giardia* at the collection sites. The locations in the region where the environmental and clinical samples were collected share the same drainage basins and were strategically selected to aid in the identification of contamination events and the detection of relationships among samples of different origins. No clinical information was obtained for the isolates.

### Clinical samples

*Giardia* spp. cysts were obtained from the feces of humans, dogs, cats and a calf. The cysts from the human samples were obtained from children at a day-care center in the city of Campinas and from patients at two hospitals: the University of Campinas Clinic Hospital and the Ouro Verde Hospital both of which are located in Campinas. The isolates obtained from dogs and cats were provided by the Animaltec Veterinary Clinic in the city of Campinas. The calf sample was obtained from São Francisco Farm in Amparo, located 37 km northeast of Campinas in the state of São Paulo. The samples were labeled and grouped according to their origin. The hospital samples were labeled “HC samples”, the day-care samples were labeled “DC samples”, and the clinical samples from non-human hosts were labeled “VET samples”.

### Ethics statement

The ethical aspects related to the acquisition of human clinical samples were approved by the ethics committee of the Faculdade de Ciências Médicas - Unicamp (251-2009). Written informed consent was obtained from legal guardians of all participants. No specific permissions were required for isolated *Giardia* cysts provided by our collaborators (VET and HC isolates). The *Giardia* cysts obtained from dogs and cats were provided by Edson Golçalves from the Animaltec Veterinary Clinic in the city of Campinas, with verbal consent of the owners, in accordance with the purposes of the research. The calf sample was obtained from São Francisco Farm in Amparo (22°42'04" S and 46°45'52" W) This sample was provided by Luciana Urbano do Santos, with consent of the owner, in accordance with the purposes of the research. Water samples were collected from hospital sewage at the University of Campinas Clinic Hospital, effluent from the Samambaia Waste Water Treatment Plant (SWWTP) (22°56′00″ S and 47°00′00″ W), a water abstraction site in Campinas (Atibaia River), the Proença urban stream (22° 53′ 16″ S and 47° 02′ 62″ W) and the Serafim urban stream (22° 53′ 20″ S and 47° 02′ 92″ W), which merge and form the Anhumas River, where another sample was collected (22° 52′ 80″ S and 47° 02′ 32″ W). No specific permissions were required for the environmental locations where the samples were collected. The field studies did not involve endangered or protected species.

### Environmental samples

Water samples were collected from hospital sewage at the University of Campinas Clinic Hospital, effluent from the Samambaia Waste Water Treatment Plant (SWWTP), a water abstraction site in Campinas (Atibaia River), and the Proença and Serafim urban streams, which merge and form the Anhumas River, where another sample was collected. These samples were labeled “ENV samples”.

### Parasitological techniques

A small aliquot obtained from the original suspension of cysts was re-examined for *Giardia* cysts using centrifugal flotation in saturated zinc sulfate [36] followed by microscopy. Following reconfirmation, the positive samples were purified on a sucrose gradient and concentrated. Antibiotics and a fungicide were subsequently added according to the original purification protocol [37].

The environmental samples were collected in bottles of different volumes, depending on the origin of the sample, and filtered (4 l/min), using a sterile mixed cellulose ester membrane with a diameter of 47 mm and a nominal porosity of 3 µm [19]. The samples were eluted by scraping the membrane with a smooth-edged plastic loop and rinsing the membrane with a 0.1% Tween 80 elution solution (RM method) [38]. The eluates were then centrifuged (1,050×*g*; 15 min), and the pellet was washed, centrifuged again and diluted in 1.5 ml of water from a 0.22 µm water filtration system (MilliQ, Millipore, Brazil). The only exception was the raw sewage sample obtained from sewage from the University of Campinas Clinic Hospital, which was concentrated via centrifugal sedimentation [39]. The pelleted samples were processed through immunomagnetic separation (IMS) using the Dynabead GC-Combo Kit followed by chemical dissociation according to the manufacturer's instructions (Dynal Biotech, Oslo, Norway). The positive samples were further confirmed using a direct immunofluorescence assay (DFA) according to the manufacturer's protocol. Briefly, 5 µl of each concentrated sample was fixed in a well slide with methanol, and fluorescein isothiocyanate-conjugated monoclonal antibodies specific for cell wall antigens from Merifluor (*Cryptosporidium*/*Giardia* Test Kit, Meridian Bioscience Inc., Cincinnati, OH, USA) were added. The cysts were identified based on their similar sizes, shapes and patterns/intensities of immunofluorescent staining using a Nikon (50i) microscope with a 450–490 nm excitation filter and a 520 nm barrier filter. The positive samples were stored in microcentrifuge tubes and frozen in liquid nitrogen to preserve their cellular integrity.

### Molecular methods

#### DNA extraction

DNA was extracted from clinical and environmental samples using the ZR Fungal/Bacterial DNA Kit (Zymo Research). Extraction was performed according to the original protocols provided by the manufacturer. Qualitative and quantitative estimation of each sample was performed via gel electrophoresis in a 1% agarose gel and with a NanoDrop 8000 Spectrophotometer (Thermo Scientific), respectively.

#### Molecular identification and genotyping of G. duodenalis assemblages

All of the extracted samples were analyzed through the amplification of fragments of the beta-giardin gene (*bg*) to confirm the presence of *Giardia*. The reactions were performed using the same reagent concentrations and thermal cycling conditions previously described with the exception of the reaction volume of 25 µl [40]. A negative control consisting of the reaction mixture without DNA was included in each run. The reactions were performed in a C1000 Thermal Cycler (BioRad) and the amplified DNA fragments were detected through gel electrophoresis in a 3% agarose gel and visualized using the Gene Genius Bio Imaging System (Syngene) after staining with ethidium bromide.

Fragments consisting of 530 bp of the triose phosphate isomerase gene (*tpi*), 753 bp of the beta-giardin gene (*bg*) and 218 bp of the glutamate dehydrogenase gene (*gdh*) were individually amplified. These markers are single-copy genes and are unlinked in the *G. duodenalis* genome [41]. Each isolate was characterized using nested *tpi* PCR, nested *gdh* PCR and *bg* PCR [42]–[44], performed according to the original protocols, with the addition of 400 ng/ µl of bovine serum albumin (BSA) in the first reaction [45]. The VET samples were analyzed with C- and D- specific *tpi* primers [46]. Following the amplification reaction, the PCR products were purified using the QIAquick PCR Purification Kit (QIAGEN) and sequenced using the ABI Prism Big Dye Terminator Cycle Sequencing Kit, version 3.1 (Applied Biosystems). The sequencing reactions were analyzed on an ABI PRISM 3500 Genetic Analyser xL (Applied Biosystems) and all of the sequences were obtained through direct sequencing in both directions a minimum of four times. The sequences were edited using CLC Main Workbench v6.8.1 (CLC Bio.) and were aligned with reference sequences using ClustalX [47]. A set of isolates with sequences that presented double peaks was cloned into the PGEM T-Easy vector (Promega Corporation). Following the transfection of *Escherichia coli* XL1-blue, 50 recombinant clones were selected for DNA sequencing.

Phylogenetic reconstruction and analysis of genealogical relationships were implemented to characterize the sequences obtained in this study based on a comparison with previously characterized sequences. Reference *bg*, *gdh*, and *tpi* sequences from all of the major genetic assemblages of *G. duodenalis* were obtained from GenBank (Table S1 in S1 File). The genotyping of each isolate was performed at four different levels of resolution. Each isolate was assigned to a specific assemblage, followed by the characterization of sub-assemblages through phylogenetic analysis. The heterogeneous profiles identified within each sub-assemblage were described as subtypes. Subtypes from the same isolate obtained for different loci were combined to generate multilocus genotypes.

#### Accession numbers of the nucleotide sequences

The accession numbers and the corresponding assemblages of the sequences obtained from the GenBank database are displayed in Table S1 in S1 File. The new sequences generated in this study have been deposited in GenBank under accession numbers JN116442-JN116504, KM495697-KM495727 and KF922892-KF923021. The accession numbers are also displayed in Tables S2, S3, S4 and S5 in S1 File.

### Phylogenetic analysis and genealogical relationships among sequences

#### Phylogenetic analysis

Neighbor-joining (NJ) and maximum likelihood (ML) analyses were performed using MEGA, version 5.05 [48]. The nucleotide substitution model that best fit the data was selected following analysis using jModelTest [49]. The analyses were conducted using the Tamura-Nei93 distance [50] with gamma correction, which assumes inequality in base frequencies and rate variation among sites. A bootstrap phylogeny test was performed with 10,000 replicates.

The Bayesian phylogenetic analysis was performed with MrBayes software version 3.1.2 [51] using the GTR model with gamma correction, which is the available model that is most similar to Tamura-Nei93. The starting trees were random, Markov chains were run for 6,000,000 iterations, and the trees were sampled every 500 iterations. Bayesian posterior probabilities were calculated using a Markov chain Monte Carlo sampling technique. Subtypes obtained from three loci were combined to define multilocus genotypes (MLGs). Isolates showing mixed assemblage profiles or overlapping nucleotides were not included in the multilocus analysis. The Bayesian inferred trees were visualized with TreeView X [52].

#### Estimation of genealogical relationships

The aligned sequences were converted to the NEXUS format [53]. The genealogical relationships between sequences were estimated using TCS software [54], which collapses identical sequences into unique haplotypes and then generates a cladogram estimate. Missing data were not considered in the haplotype analyses. An absolute distance matrix was calculated for all pairwise comparisons of haplotypes. The probability of parsimony was calculated for pairwise differences until the probability exceeded 0.95. DNAsp software v5.10 [55] was used to generate an Arlequin data file. Arlequin software was employed to calculate haplotype diversity at the intra-population level [56].

## Results

### Detection of positive samples using parasitological methods

All of the surveyed environmental and clinical sites (Fig. 1) provided samples that were positive for *Giardia* spp.; in total, 91 positive samples were obtained. Among the human clinical samples, 51 positive samples were provided by hospitals (HC group) and 28 positive samples from children were provided by the day-care center (DC group). Six positive samples were obtained from non-human hosts (VET group): three from dogs (VET01, VET02 and VET03), two from cats (VET04 and VET05) and one from a calf (VET06). Among the environmental samples (ENV group), one positive sample was obtained from the SWWTP (ENV01), one from the water abstraction site of the Atibaia River (ENV02) and one from hospital sewage from the University of Campinas Clinic Hospital (ENV06). Two positive samples were identified from urban streams: one from the Proença stream (ENV03) and one from the Serafim stream (ENV04). These streams merge to form the Anhumas River, where another positive sample was identified (ENV05). In total, 87% (79/91) of the obtained *Giardia* cysts came from human clinical specimens, 6.5% (6/91) were from non-human specimens and 6.5% (6/91) were from environmental samples.

### Molecular analyses

#### Molecular identification of *G. duodenalis*

The results of the amplification of the fragments of the three genes were used to confirm the presence of *Giardia* in all of the samples. Consensus analysis of the complementary molecular methods (*gdh, tpi,* and both approaches for *bg*) confirmed 97% (88/91) of the samples to be positive. Amplification reactions targeting two different fragments of the *bg* gene [40] confirmed 51% (46/91) positivity. The amplification rates (followed by sequencing) of the three other markers used were 89% (81/91), 69% (63/91) and 49% (45/91) for the *tpi*, *gdh* and *bg* genes, respectively. Three samples (HC03, HC05 and HC26) were positive by DFA but repeatedly negative in all of the amplification reactions.

#### Molecular genotyping at the beta-giardin (*bg*) locus

Among the 45 *bg* sequences generated, assemblage A was identified in 34 isolates: 15 from the HC group and 19 from the DC group. Nine assemblage B isolates were identified in the HC group. One assemblage D isolate and one assemblage E isolate were found in the VET group in samples from a dog (VET01) and a calf (VET 06), respectively. No sequence data were obtained for this marker in the ENV group. The distribution of each genetic assemblage within each source of contamination is presented in Table 1.

**Table 1:** Distribution of genetic assemblages as the percentage within each source.

Assemblage	Sources of Infection*			
	HC (%)	DC (%)	VET (%)	ENV (%)
A	44.6	47.5	22.2	14.3
B	**46.4**	**52.5**	**33.3**	**57.1**
C	9	0	22.2	14.3
D	0	0	16.7	14.3
E	0	0	5.6	0

Each source of samples was examined to identify the genetic assemblages that contributed most to the isolates. The relative distribution considers all of the genes and includes the results of mixed assemblages. Bold numbers indicate the highest percentage per column. * Hospital group (HC), day-care center group (DC), veterinary group (VET) and environmental group (ENV).

#### Molecular genotyping at the triose phosphate isomerase (*tpi*) locus

Among the 81 *tpi* sequences obtained through the reaction with generic primers, assemblage A was identified in 20 HC, five DC and three VET isolates. Forty-seven isolates (21 from HC, 20 from DC, two from VET and four from ENV) were classified as belonging to assemblage B, which was the most prevalent assemblage at this locus. Five assemblage C isolates from the HC group and another one from the ENV group were identified. When *tpi* primers specific for assemblages C and D were used, sequencing revealed assemblage D in two isolates (VET01 and VET05) and assemblage C in an additional isolate (VET02), both from the VET group. Remarkably, these three samples had previously been characterized as belonging to different assemblages using the generic *tpi* primers.

#### Molecular genotyping at the glutamate dehydrogenase (*gdh*) locus

At the *gdh* locus, five different assemblages were identified. Assemblage A was detected in 28 isolates (20 from HC, seven from DC and one from the ENV group). Assemblage B was found in 29 isolates (17 from HC, 11 from DC and one from the ENV group). Four isolates were characterized as belonging to assemblage D (three from VET and one from the ENV group). The isolate obtained from the calf was genotyped as belonging to assemblage E (VET06), and one isolate obtained from a dog was characterized as assemblage C (VET03). Table S6 in S1 File presents the comparison of each source of contamination and its distribution within each genetic assemblage.

#### Combined genotyping results at the assemblage and sub-assemblage levels

A total of 192 sequences were generated in this study: 63 for the *gdh* gene, 45 for the *bg* gene, 81 for the *tpi* gene and three additional sequences through assemblage-specific *tpi* PCR. Sequence data were obtained for three genes from 37 samples, for two genes from 29 samples and for one gene from 22 samples. In the sequenced hospital samples (n = 48), assemblages A (42%), B (42%) and C (6%) were identified. Contrasting assemblage results between markers were found in five isolates. All assemblage A isolates from the HC group corresponded to sub-assemblage AII. Among the assemblage B isolates from this group, only one (HC-33) was characterized as BIII, whereas the remainder were BIV. Similar results were observed in the DC group, in which all assemblage A isolates were characterized as AII. Five isolates were genotyped as BIV and four as BIII. Twelve samples presented different assemblage results between markers. Many genetic assemblages were found in the VET group, in which most of the isolates from dogs and cats and that from the calf were characterized as belonging to host-adapted assemblages (C and D for dogs and cats and E for the calf). However, the sequences generated using the generic *tpi* primers were characterized as belonging to assemblages with zoonotic potential (S1 Fig.). Most of the isolates from the ENV group exhibited assemblages with zoonotic potential even though assemblages C and D were identified at the water abstraction point of the city (ENV02) and in the Anhumas River (ENV05), respectively. The genotyping results for each sample are presented in Tables S2, S3, S4, and S5 in S1 File. The identified assemblages were plotted on the physical map close to their respective collection sites (Fig. 1).

#### Mixed assemblages and heterogeneous sequencing profiles

All of the surveyed groups presented at least one sample in which the assignment to a specific assemblage was inconsistent when two or more different loci were compared. Mixed assemblages in an individual isolate were identified in 26% (23/88) of all sequenced samples. When considering only isolates that were sequenced with at least two different markers or were cloned, the proportion of mixed assemblages increased to 34% (23/66). In the HC group, 12% (6/48) of the isolates were identified as showing this pattern. Two human isolates presented both assemblages A and C, and another three isolates presented mixtures of A and B. One isolate (HC-07) exhibited assemblages BIII and BIV. Within the DC group, 39% (11/28) of the isolates presented mixed assemblages, all of which involved assemblages A and B. The highest prevalence of mixed assemblages was found in the VET group, in which 83% (5/6) of the samples were characterized as displaying different assemblages. This prevalence increased to 100% when considering only the samples genotyped with at least two markers. In two cases, more than two assemblages were observed in individual VET samples (VET02 and VET04). In the ENV group, only isolate ENV-05 presented a mixed assemblage (BIV and D), even though most of the samples (4/6) were successfully sequenced with only one marker. Of the 22 isolates with mixed assemblages identified, 64% (14/22) involved assemblages A and B. Three isolates presented assemblages B and D, two presented A and C, and one presented A and E. Table 2 provides the genetic assemblages of each isolate in which mixed assemblages were identified.

**Table 2:** Molecular characterization of isolates presenting mixed assemblage infections.

Isolate	*gdh*	*bg*	*tpi*	*tpi*-specific	Source
HC02	A	-	C	-	Hospital
HC07	-	BIII	BIV	-	Hospital
HC09	-	AII	BIV	-	Hospital
HC13	A	-	C	-	Hospital
HC21	B	-	AII	-	Hospital
HC23	B	-	AII	-	Hospital
DC01	B	AII	BIV	-	Daycare
DC02	-	AII	BIV	-	Daycare
DC03	B	AII	BIV	-	Daycare
DC05	B	AII	BIV	-	Daycare
DC08	-	AII	BIV	-	Daycare
DC11	-	AII	BIII	-	Daycare
DC14	-	AII	BIV	-	Daycare
DC19	B	AII	BIV	-	Daycare
DC20	B	AII	BIV	-	Daycare
DC22	B	AII	BIV	-	Daycare
DC23	-	AII	BIII	-	Daycare
VET01	D	D	BIV	D	Veterinary
VET02	D	-	AII/AI/C*	C	Veterinary
VET04	-	-	AII/BIII/BIV*		Veterinary
VET05	D	-	BIV	D	Veterinary
VET06	E	E	AII	-	Veterinary
ENV05	D	-	BIV	-	Environment

Occurrence of mixed assemblages in the isolates based on the molecular characterization of sequences of the glutamate dehydrogenase (*gdh*), beta-giardin (*bg*) and triose phosphate isomerase (*tpi*) genes. Twenty-three of the 88 sequenced samples presented mixed assemblages. * Sequences were obtained after molecular cloning.

Overlapping nucleotides in the chromatograms were verified in a considerable number of sequences. Thirty-seven percent (22/63) of the *gdh* sequences presented double peaks, whereas 27% (11/45) of the *bg* sequences and 26% (21/84) of the *tpi* sequences presented this pattern. In all of the assemblages, at least one isolate exhibited double peaks in the chromatogram. Most of the samples in which double peaks were observed were characterized as belonging to assemblage B. To assure reproducibility, overlapping nucleotides were only considered when the same pattern was observed in different sequencing reactions for the same sample. A complete list with all isolates that presented double peaks is displayed in Table S7 in S1 File.

Following the cloning of fragments with double peaks, additional sequences were detected: three new sequences were identified from the VET04 sample (one BIV, one BIII and one C) and three from the VET02 sample (two C and one AI). Three different subtypes were identified from the HC43 sample (all BIV). The sequences from HC51(*gdh*), VET06(*tpi*), DC04(*tpi*), DC05(*tpi*) and ENV06(*gdh*) presented only one haplotype phase.

#### Subtypes

Subtypes could be determined only when double peaks were not identified at any position. At the *bg* locus, 33 of the 45 (73%) sequenced isolates exhibited no double peaks. These 33 isolates corresponded to 13 subtypes, including nine new subtypes and four previously reported subtypes. The novel subtypes were represented by one isolate each (Table S8 in S1 File). At the *gdh* locus, 40 of 63 (63%) isolates presented no overlapping nucleotides in the chromatogram. Eight different subtypes were observed, two of which corresponded to novel subtypes. The most common subtype was found in 21 isolates (Table S9 in S1 File). Among the 84 sequences obtained at the *tpi* locus, 62/84 (74%) exhibited no double peaks. Thirty different subtypes were detected, 21 of which were new. The most common subtype was observed in 17 isolates (Table S10 in S1 File).

#### Genealogical relationships

A similar proportion of haplotypes was found when different molecular markers were compared. As the focus of this analysis was not the isolates but the haplotypes, isolates with overlapping nucleotides were included. In total, 20, 49 and 24 different haplotypes were identified for the *bg, tpi* and *gdh* genes, respectively (Table 3).

**Table 3:** Distribution of different haplotypes from each genetic assemblage within each gene.

Assemblage	*bg*	*tpi*	*gdh*
A	**10**	11	4
B	8	**27**	**15**
C	-	10	1
D	1	1	3
E	1	-	1
Total	20	49	24

The different haplotypes identified in each genetic assemblage are presented for the glutamate dehydrogenase (*gdh*), beta-giardin (*bg*) and triose phosphate isomerase (*tpi*) genes. Bold numbers indicate the assemblage that contributed most to the haplotypes.

**Figure 2:**
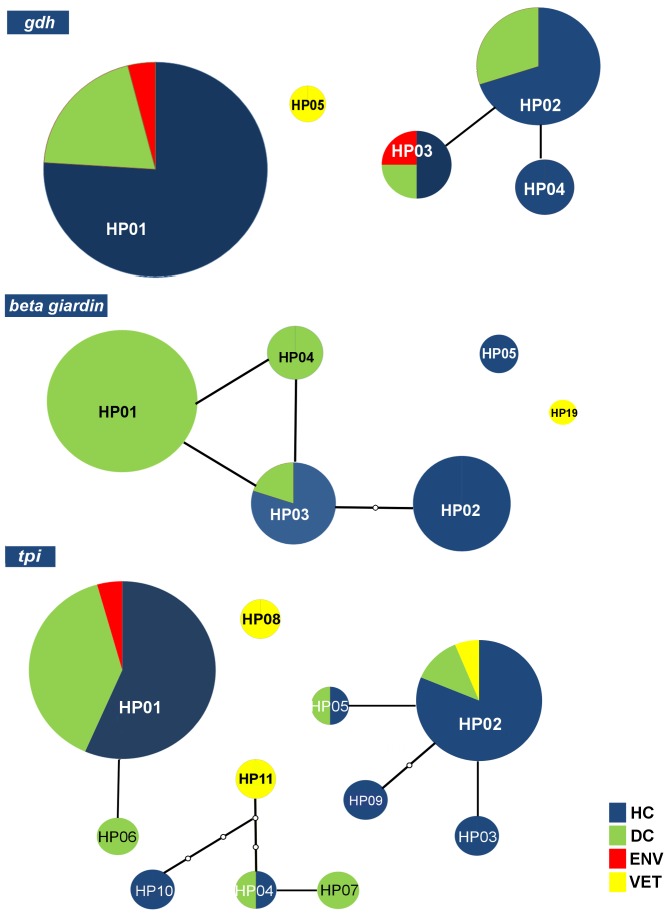
Networks of *G. duodenalis* haplotypes within the *gdh, bg, and tpi* genes. The relative frequencies of the main haplotypes from the day-care center (green), hospital clinical (dark blue), veterinary (yellow) and environmental (red) samples generated from a statistical parsimony network. The sizes of the circles are proportional to the haplotype frequency. The lines connecting each haplotype represent one mutation, and small white circles represent inferred haplotypes that were not actually observed. Haplotypes that are not connected to others exhibited more steps (or mutations) than the connection limit (95% confidence).

Considering all of the examined molecular markers, assemblage B was the most common, with 50 haplotypes, followed by assemblage A, with 25. Assemblages C, D and E presented 11, five and two different haplotypes, respectively. Most haplotypes from the *tpi* and *gdh* genes were characterized as belonging to assemblage B. At the *bg* locus, there were similar proportions of A and B. The relative frequencies of each source within the main haplotypes are presented in Fig. 2.

Many haplotypes were found in the four groups. The HC group exhibited 48 different haplotypes, most of which were from assemblage B isolates. A comparable result was observed in the DC group, in which 31 different haplotypes were found (61% from assemblage B), and in the ENV group, in which 62.5% (5/8) of the haplotypes were from assemblage B. In contrast, the VET group presented a balanced distribution of haplotypes in many assemblages (Table 4). Haplotype variation was detected in almost all of the environmental samples. A complete list of haplotypes is provided in Tables S11, S12 and S13 in S1 File.

**Table 4:** Distribution of haplotypes from each genetic assemblage within each source.

Assemblage	Sources of Infection*			
	HC	DC	VET	ENV
A	14	12	4	1
B	**29**	**19**	3	**5**
C	5	-	**5**	1
D	-	-	4	1
E	-	-	2	-
Total	48	31	18	8

The haplotypes identified in each genetic assemblage are presented for each gene. Bold numbers indicate the assemblage that contributed most to the haplotypes. * Hospital group (HC), day-care center group (DC), veterinary group (VET) and environmental group (ENV).

The sequences of the most frequent haplotypes were compared to sequences available in the GenBank database. These sequences shared identity with several sequences identified in humans, animals and even environmental samples. The only exception was the HP01-*bg* haplotype, which contained 13 identical sequences (100% from the DC group) that did not exactly match any sequence previously deposited in GenBank and was thus considered a novel sequence (Table S14 in S1 File).

#### Genetic diversity

Haplotype diversity was evaluated in the four groups. The DC group exhibited considerable haplotype diversity for the *gdh* (0.91±0.05) and *tpi* (0.85±0.06) genes. The *bg* gene revealed the lowest rate (0.34±0.14). The HC group presented rates of 0.71±0.07, 0.85±0.03 and 0.92±0.05 for the *gdh*, *tpi* and *bg* genes, respectively. The ENV and VET groups also presented high levels of haplotype diversity, but this result might be an artifact due to the small sample sizes of these groups.

#### Multilocus genotyping

Only 13 samples from the 37 isolates sequenced at the three loci showed no double peaks and presented complete agreement across all genes. The subtypes that were sequenced at all three loci and shared the same assemblage were grouped into 7 MLGs for assemblage A (labeled as BRA1-BRA7), and 3 MLGs for assemblage B isolates (labeled as BRAB1-BRAB3). The MLGs defined for assemblage A (Table 5) corresponded to assemblage AII, and the MLGs defined for assemblage B (Table 6) all corresponded to assemblage BIV. All of these MLGs were obtained from HC and DC isolates. All of the MLGs defined for assemblages A and B and their mutations for each gene are displayed in Tables S15 and S16 in S1 File.

**Table 5:** Multilocus genotypes (MLGs) in *G. duodenalis* assemblage A isolates.

MLG	Isolate	*bg*	*gdh*	*tpi*	Sub-assemblage
		Subtypes			
BRA1	HC31/HC48	bg-1 (kc8)	gdh-1	tpi-1 (Ad2)	AII
BRA2	DC15	bg-2	gdh-1	tpi-1 (Ad2)	AII
BRA3	DC27/HC44	bg-2	gdh-1	tpi-2	AII
BRA4	DC28	bg-2	gdh-1	tpi-3	AII
BRA5	HC10/HC49	bg-3	gdh-1	tpi-1 (Ad2)	AII
BRA6	HC12	bg-4	gdh-1	tpi-4	AII
BRA7	HC27	bg-5	gdh-1	tpi-1 (Ad2)	AII

Subtypes of the sub-assemblage AII were grouped into seven different multilocus genotypes.

**Table 6:** Multilocus genotypes (MLGs) in *G. duodenalis* assemblage B isolates.

MLG	Isolate	*bg*	*gdh*	*tpi*	Sub-assemblage
		Subtypes			
BRAB1	HC33	bgB-1	gdhB-2	tpiB-2	BIV
BRAB2	HC46	bgB-2	gdhB-1 (Ad7)	tpiB-1 (Ad19)	BIV
BRAB3	HC47	bgB-2	gdhB-3	tpiB-1 (Ad19)	BIV

Subtypes of the sub-assemblage BIV were grouped into three different multilocus genotypes.

**Figure 3:**
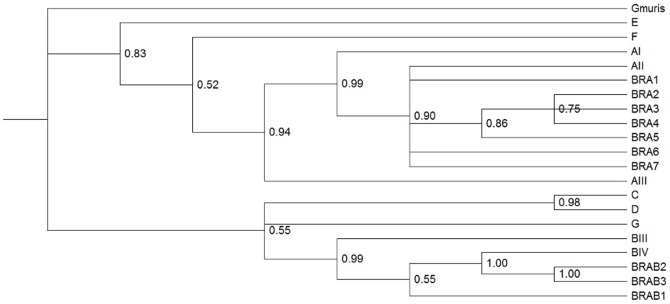
Phylogenetic relationships of the concatenated *bg, tpi, and gdh* genes of *G. duodenalis*. Phylogenetic relationships of *G. duodenalis* with Bayesian posterior probabilities using a Markov chain Monte Carlo sampling technique. Markov chains were run for 6,000,000 iterations and the trees were sampled every 500 iterations. The GTR model was used with gamma correction. Sequences from *G. muris* were employed used as an outgroup.

The trees obtained from the concatenated *tpi*, *bg* and *gdh* sequences presented similar results in comparison with the phylogenetic analyses of individual genes and indicated that no recombination events had occurred among these isolates. The topologies observed within different *Giardia* assemblages confirmed the monophyly of assemblages A and B (Fig. 3). Multilocus genotyping was also performed for samples in which sequences from two genes were obtained. This analysis allowed us to characterize three isolates from assemblage A (Table S17 in S1 File) in the proposed MLGs and to identify four new MLGs from assemblage B (Table S18 in S1 File), including one isolate from an environmental sample. The genetic differences among all genetic assemblages are displayed in Tables S19, S20 and S21 in S1 File.

## Discussion

### *G. duodenalis* contamination in the metropolitan region of Campinas

Protozoan contamination has regularly been documented at important environmental sites in the metropolitan region of Campinas. Previous studies have demonstrated the occurrence of *Giardia* spp. and *Cryptosporidium* spp. in superficial raw water from the Atibaia River [19], waste water from an effluent treatment plant [57], activated sludge samples from a sewage treatment plant [22], and the intake pipe of a water treatment plant in Campinas [58]. A recent study recognized the metropolitan region of Campinas as presenting the highest risk of *Giardia* infection among four densely populated regions of Sao Paulo State, Brazil [21]. Likewise, many clinical studies in the same region have reported *Giardia* infections among children at day-care centers [59] as well as the users of public health services [60], [61], and schools [62] and inhabitants of farms [63]. The great majority of studies only obtained data on the species level based on the morphology of the parasites. Moreover, there is a remarkable lack of studies that correlate environmental contamination with clinical infections in the same region.

Our results demonstrate that there is persistent contamination at important environmental sites in the city of Campinas, such as in streams in the downtown area and rivers (Anhumas River and Atibaia River). This contamination is relevant for people who live in this region. The Atibaia River is impacted by discharge from domestic and industrial sewage and contamination from agricultural sources [58]. The contaminated effluent from the Samambaia Waste Water Treatment Plant represents another source of pathogenic organisms for this river. This effluent is discharged into the Pinheiros Stream, which flows to the Atibaia River. This contamination is important to public health, as several cities use the water of this river for drinking water. The clinical samples obtained in this study (day-care and hospital samples) are discharged into the Anhumas River, which is another tributary of the Atibaia River. These sources of contamination certainly contribute to the high risk of *Giardia* infection assigned to this metropolitan region [21].

### Prevalence of each genetic assemblage in environmental and clinical isolates

Assemblages A and B were the most common in the human clinical samples. These results were expected because people are predominantly infected with these *Giardia* assemblages. In contrast, assemblage C was identified in five human hospital samples. Although this assemblage has been previously reported in human samples [34], [64], this result can be considered unusual because assemblage C is generally identified in dogs, cats, coyotes and wolves [34]. The detected assemblage C subtypes did not match the other subtypes available in the databases, with the exception of the HC13 isolate, for which an identical sequence was identified in a dog from Sweden [46].

The isolates sequenced from non-human clinical samples belonged mainly to host-adapted assemblages. However, the isolates from this group could be sequenced using the generic *tpi* marker, revealing the presence of assemblages with zoonotic potential in 83% (5/6) of the veterinary samples, leading us to conclude that these assemblages may present a risk to public health. Although dogs, cats and livestock are usually infected with host-adapted *G. duodenalis* assemblages [12], [18], these animals can act as potential reservoirs for zoonotic transmission, which might serve as another source of infection for people living in this region.

In the present study, most of the environmental samples were characterized as belonging to assemblages A and B, including the waste water sample. Similar results have been obtained at other waste water treatment plants in other countries, such as China [65], the United States [66] and France [67]. These results suggest that anthroponotic transmission is important in these environmental sources, although the zoonotic potential of these samples must be considered. Assemblage D was also observed in the Anhumas River, and assemblage C was identified at the water abstraction site of the city. These findings suggest that there are other sources of contamination at these environmental sites. The identification of *Giardia* cysts in water supplies may contribute substantially to the protection of public health in the investigated areas [68].

### Mixed assemblages and heterogeneous sequence profiles

In the present work, mixed assemblage isolates were observed in the human hospital samples (12%) and a remarkably high rate of such assemblages (43%) was noted in children from the day-care center. It is also noteworthy that in all of the mixed assemblages obtained from the day-care center, all *bg* sequences were identified as assemblage AII, whereas the other markers indicated assemblage BIV or BIII. This difference may be due to mixed infections showing preferential amplification of single genotypes over others at a specific locus [69]. Mixed assemblage isolates have been frequently reported in studies that make use of more than a single molecular marker [34], [69]–[71]. The finding that most of the mixed assemblages identified in this study were from assemblages A and B can be explained by the overrepresentation of human samples, which were predominantly assigned to these assemblages.

Despite the small number of veterinary samples examined, mixed assemblages were found in all of the veterinary samples sequenced with at least two markers. This result suggests that there must be high endemicity of *Giardia* in these animals and that they likely continue to re-infect themselves due to contact with the feces of other infected animals. Further studies are required to determine the prevalence rates of *Giardia duodenalis* and the most common *G. duodenalis* assemblages in both household and kennel animals in this metropolitan region.

Overlapping nucleotides were identified at a high frequency with similar rates among all three markers. Similar results were found in lambs from Spain, in which the frequency of mixed templates reached 44% for the *tpi* gene [72]. A study of human and animal samples conducted in Italy, Africa and Croatia detected rates of overlapping nucleotides of 36%, 50% and 75% in the sequences of the *bg*, *tpi* and *gdh* genes, respectively [73]. Several studies have reported a high percentage of double peaks in the chromatograms in various animal species, including humans [46], [74], [75].

Three cloned samples in which more than one haplotype had been previously detected, presented different subtypes in their clones. Two samples (VET02 and VET04) exhibited different genetic assemblages in their clones. Sample HC43 displayed different subtypes, all from the same genetic assemblage. After sequencing of the clones, these sequences were found to present more polymorphisms than had been detected in the double peaks. These results suggest that most sequences showing double peaks previously identified at specific sites most likely represent mixed assemblages, rather than allelic sequence heterozygosity.

The uncertainty regarding the origin of the mixed assemblages and/or mixed templates is due to the difficulty associated with determining whether the mixed profile arose through genetic exchange, allelic polymorphisms, mixed/concurrent infections or a combination of all of these events. Higher rates of mixed assemblages were observed in the DC and VET groups. Because there is close contact among children at day-care centers and among infected dogs and cats in the streets, these results likely represent mixed infections.

### Haplotype diversity and zoonotic potential

The main importance of the haplotypes analysis lies in the observation that the number of isolates found in each genetic assemblage is not necessarily followed by a proportional number of haplotypes depending on the genetic diversity of each group of samples or the molecular markers used. In the present study, the haplotype analysis of the *tpi* and *bg* genes indicated that there was higher variability in samples of assemblage B, which is consistent with previous studies investigating sequence polymorphisms in assemblage B compared with assemblage A [76], [77].

A considerable proportion of different haplotypes was found at all of the sources from which positive samples were obtained. The haplotype variability observed in the hospital samples (48 haplotypes) can be explained by both the different origins of people who attend these hospitals and the overall high genetic variability of the human samples classified into assemblages A and B. Patients at these hospitals come not only from the metropolitan region but also from the state of São Paulo. The pronounced variability in the HC group most likely reflects the haplotypes of the entire state. Further molecular surveillance studies are required to verify the extent of hospital sewage contamination in the water sources for the metropolitan region, as this sewage may be introducing new *Giardia* haplotypes into environmental sites in this region.

The haplotype variability detected in the samples from the day-care center (31 haplotypes) may be a consequence of the multiple sources of infection observed at this location combined with the fact that children attending this center live in conditions of socioeconomic vulnerability and experience unsanitary crowding conditions. Several potential sources of zoonosis (including dogs and cats) have been observed in the vicinity of the day-care center, and these species may have served as reservoirs for zoonotic transmission. The presence of some haplotypes with high frequencies suggests that these haplotypes may have been introduced into the day-care center population through person-to-person transmission.

Analyses of the VET and ENV groups revealed a regular distribution of haplotypes with similar frequencies. The VET group consists of different animals with infections that likely originated from different sources. Our results for the ENV group suggest that many different haplotypes are present at different environmental sites. These sites are vulnerable to many sources of contamination, such as hospital and domestic sewage, animal feces and agricultural and industrial sewage. All of these sources may contribute to the observed haplotype variability, and further studies are necessary to monitor the most common haplotypes of *Giardia* at each environmental site.

Although a considerable number of haplotypes were identified, most of the samples originated from only a few haplotypes. Thus, only a small proportion of the isolates were responsible for the large number of haplotypes detected. The observation of a particular haplotype in different groups suggests potential relationships among these groups, which is consistent with the previously demonstrated interconnections of the collection sites. Determination of whether one group is contaminating all of the others or if they are all vulnerable to the same sources of contamination, will require further molecular surveillance studies.

### Characterization of the genetic profile of G. duodenalis in the metropolitan region via multilocus genotyping

In the present study, 10 different MLGs were identified in the isolates from the hospitals and day-care center. With the exception of the BRA1 MLG, all other MLGs corresponded to new profiles. The identified MLGs may contribute to future studies designed to establish a genetic profile of the most common genotypes found in this region. A secondary analysis, including isolates that lacked one of the three gene sequences, allowed us to identify new profiles, including a new MLG originating from an environmental sample. Characterization of environmental isolates should play an important role in future studies given the absence of information about contributors to environmental contamination [73].

## Final remarks

In conclusion, in addition to *Giardia* contamination at important environmental sites and infections in non-human hosts, our data demonstrate that most of the *Giardia* isolates obtained in this work can be considered to show zoonotic potential. There was a high degree of genetic diversity in the isolates obtained from both clinical and environmental samples, suggesting that multiple sources of infection are responsible for the detected contamination events. The detection of different assemblages and subtypes (after molecular cloning) in isolates with and without double peaks suggests that most studies likely underestimate the real genetic diversity of this parasite.

Our results, together with those of previous studies reporting environmental contamination and prevalence of *Giardia* above the limits tolerated by the United States Environmental Protection Agency (USEPA) [19], [21], [22], indicate a notable risk for human infection by *Giardia* in this region.

Future molecular surveillance studies of *G. duodenalis* and other waterborne protozoans are essential to elucidate the genetic profile of the distribution of these parasites in this region. Water sources, waste water effluents, water treatment plants, sewage treatment plants, hospitals, day-care centers, and public kennels are some key potential study sites. Analyses of population genetic structure conducted with high-resolution tools are needed to elucidate additional features of the origin and dissemination of the parasite [78]. Complementary approaches are necessary to detect mixed sequence profiles and rare haplotypes to avoid underestimation of the genetic diversity of *Giardia*, such as a) molecular cloning and sequencing of amplified fragments, b) the use of assemblage-specific primers, and c) high-throughput next-generation sequencing. The multilocus genotype approach presented herein can be considered to represent an initial step in the characterization of *Giardia* diversity in the metropolitan region of Campinas and can help to track sources of contamination associated with this waterborne protozoan. Furthermore, to prevent the spread of waterborne diseases, new public policies should consider not only the city but also the complex relationships among different cities that share the same metropolitan region.

## Supporting Information

S1 Fig **Phylogenetic analysis of the VET group.** Phylogenetic analysis of the *Giardia* triose phosphate isomerase (*tpi*) gene from the VET group. The alignment was generated with ClustalX and analyzed using maximum likelihood with the Tamura-Nei 93 model with gamma correction (MEGA v5.05). Trees derived using neighbor-joining produced a similar topology. Bootstrap values>50% (10,000 replicates) are shown beside each node. Accession numbers for *tpi* reference sequences are shown beside the corresponding assemblage.
(TIF)

S1 File **Table S1, Accession numbers for gene sequences obtained from NCBI.** All reference sequences used in this study from the three genes are listed in this table. **Table S2, Molecular characterization and accession numbers of isolates obtained from hospitals based on sequencing data from the** ***gdh*** **,** ***bg*** **, and** ***tpi*** **genes.** The genetic assemblage of each sequenced isolate from the HC group and its accession numbers are displayed in this table. **Table S3, Molecular characterization and accession numbers of isolates obtained from the day-care center based on sequencing data from the** ***gdh*** **,** ***bg*** **, and** ***tpi*** **genes.** The genetic assemblage of each sequenced isolate in the DC group and its accession numbers are displayed in this table. **Table S4, Molecular characterization and accession numbers of isolates obtained from veterinary samples based on sequencing data from the** ***gdh*** **,** ***bg*** **, and** ***tpi*** **genes.** The genetic assemblage of each sequenced isolate from the VET group and its accession numbers are displayed in this table. **Table S5, Molecular characterization and accession numbers of isolates obtained from environmental samples based on sequencing data from the** ***gdh*** **,** ***bg*** **, and** ***tpi*** **genes.** The genetic assemblage of each sequenced isolate from the ENV group and its accession numbers are displayed in this table. **Table S6, Distribution of each source as a percentage within each genetic assemblage.** Each assemblage was examined to identify the source that contributed the most isolates. The table presents the proportion of each source of contamination (HC, DC, VET and ENV) within each genetic assemblage. **Table S7, Samples with double peaks within each loci.** All samples that presented double peaks are listed based on sequencing data from the *gdh*, *bg*, and *tpi* genes. **Table S8, Correspondence between subtypes and sequences of the** ***bg*** **gene in the isolates.** Correspondence between subtypes of the *bg* gene and the associated isolates. Each subtype (S) presents at least one isolate. The main subtypes revealed many isolates in one subtype. **Table S9, Correspondence between the subtypes and sequences of the** ***gdh*** **gene in the isolates.** Correspondence between subtypes of the *gdh* gene and the associated isolates. Each subtype (S) presents at least one isolate. The main subtypes revealed many isolates in one subtype. **Table S10, Correspondence between subtypes and sequences of the** ***tpi*** **gene in the isolates.** Correspondence between subtypes of the *tpi* gene and the associated isolates. Each subtype (S) presents at least one isolate. The main subtypes revealed many isolates in one subtype. **Table S11, Correspondence between the haplotypes and sequences of the** ***gdh*** **gene in the isolates.** Correspondence between haplotypes of the *gdh* gene and the associated isolates. The main haplotypes presents many isolates with each haplotype. Some haplotypes showed a mixture among the different groups. **Table S12, Correspondence between the haplotypes and sequences of the** ***tpi*** **gene in the isolates.** Correspondence between the haplotypes of the *tpi* gene and the associated isolates. The main haplotypes present many isolates with each haplotype. Some haplotypes showed a mixture among the different groups. **Table S13, Correspondence between the haplotypes and sequences of the** ***bg*** **gene in the isolates.** Correspondence between the haplotypes of the *bg* gene and the associated isolates. The main haplotypes present many isolates with each haplotype. Some haplotypes revealed a mixture among the different groups. **Table S14, Comparison of each haplotype identified with sequences available in the GenBank database.** This table shows the main haplotypes obtained in this study. These haplotypes were compared with the GenBank database. Many identical sequences were identified. The haplotype HP01-*bg* was not identified in the database and corresponds to a new sequence. **Table S15, Polymorphic sites in the** ***bg*** **and** ***tpi*** **sequences among** ***G. duodenalis*** **assemblage A isolates.** This table presents all mutations that contributed to differences in each multilocus genotypes from assemblage A isolates. The *gdh* gene did not present any differences among the multilocus genotypes identified. **Table S16, Polymorphic sites in the** ***bg, tpi*** **, and** ***gdh*** **sequences among** ***G. duodenalis*** **assemblage A isolates.** This table presents all mutations that contributed to differences in each multilocus genotype from assemblage B isolates. **Table S17, Multilocus genotypes (MLGs) in** ***G. duodenalis*** **assemblage A isolates.** Multilocus genotypes obtained from assemblage A isolates in which at least two sequences were obtained. **Table S18, Multilocus genotypes (MLGs) in** ***G. duodenalis*** **assemblages B isolates.** Multilocus genotypes obtained from assemblage B isolates in which at least two sequences were obtained. **Table S19, Genetic differences in the genetic assemblages at the** ***gdh*** **locus.** All SNPs in the different genetic assemblages are displayed within *gdh* loci. **Table S20, Genetic differences in the genetic assemblages at the** ***bg*** **locus.** All SNPs in the different genetic assemblages are displayed within *bg* loci. **Table S21, Genetic differences in the genetic assemblages at the** ***tpi*** **locus.** All SNPs in the different genetic assemblages are displayed within *tpi* loci.
(DOCX)
