# Supplementary material for: Genetic Diversity of Giardia duodenalis: Multilocus Genotyping Reveals Zoonotic Potential between Clinical and Environmental Sources in a Metropolitan Region of Brazil
Source: PLoS One. 2014 Dec 23;9(12):e115489. doi: 10.1371/journal.pone.0115489 (PMC4275228; doi:10.1371/journal.pone.0115489)
Supplement: S1 File — Table S1, Accession numbers for gene sequences obtained from NCBI. All reference sequences used in this study from the three genes are listed in this table. Table S2, Molecular characterization and accession numbers of isolates obtained from hospitals based on sequencing data from the gdh , bg , and tpi genes. The genetic assemblage of each sequenced isolate from the HC group and its accession numbers are displayed in this table. Table S3, Molecular characterization and accession numbers of isolates obtained from the day-care center based on sequencing data from the gdh , bg , and tpi genes. The genetic assemblage of each sequenced isolate in the DC group and its accession numbers are displayed in this table. Table S4, Molecular characterization and accession numbers of isolates obtained from veterinary samples based on sequencing data from the gdh , bg , and tpi genes. The genetic assemblage of each sequenced isolate from the VET group and its accession numbers are displayed in this table. Table S5, Molecular characterization and accession numbers of isolates obtained from environmental samples based on sequencing data from the gdh , bg , and tpi genes. The genetic assemblage of each sequenced isolate from the ENV group and its accession numbers are displayed in this table. Table S6, Distribution of each source as a percentage within each genetic assemblage. Each assemblage was examined to identify the source that contributed the most isolates. The table presents the proportion of each source of contamination (HC, DC, VET and ENV) within each genetic assemblage. Table S7, Samples with double peaks within each loci. All samples that presented double peaks are listed based on sequencing data from the gdh, bg, and tpi genes. Table S8, Correspondence between subtypes and sequences of the bg gene in the isolates. Correspondence between subtypes of the bg gene and the associated isolates. Each subtype (S) presents at least one isolate. The main subtypes revealed many iso [file pone.0115489.s002.docx]

**Table S1.** Accession numbers for gene sequences obtained from NCBI.

|  | ***gdh*** | ***bg*** | ***tpi*** |
| --- | --- | --- | --- |
| AI | L40509 | M36728.1 | L02120.1 |
| AII | L40510 | AY072723.1 | U57897.1 |
| AIII | - | DQ648777.1 | DQ650648.1 |
| BIII | AF069059.1 | AY072726.1 | AY228628.1 |
| BIV | L40508.1 | AY072725.1 | L02116.1 |
| C | U60985.1 | JF422719.1 | AY228641.1 |
| D | U60986.2 | AY545647.1 | DQ246216.1 |
| E | AY178740 | AY072729.1 | AY655705.1 |
| F | AY178744 | AY647264.1 | AF069558.1 |
| G | AY178748.1 | EU769221.1 | EU781013.1 |
| *G. muris* | - | EF455599.1 | AF069565.1 |
| *G. ardeae* | AF069060.2 | - | AF069564.1 |
| *G. microti* | - | - | AY228649.1 |

**Table S2.** Molecular characterization and accession numbers of isolates obtained from hospitals based on sequencing data from *gdh*, *bg*, and *tpi* genes.

| **Sample** | ***gdh*** | ***GenBank*** | ***bg*** | ***GenBank*** | ***tpi*** | ***GenBank*** |
| --- | --- | --- | --- | --- | --- | --- |
| HC01 | A | JN116442 | - | - | AII | KF922892 |
| HC02 | A | JN116443 | - | - | C | KF922893 |
| HC03 | - | - | - | - | - | - |
| HC04 | - | - | - | - | C | KF922894 |
| HC05 | - | - | - | - | - | - |
| HC06 | - | - | - | - | AII | KF922895 |
| HC07 | B | JN116444 | BIII | KF922976 | BIV | KF922896 |
| HC08 | - | - | - | - | C | KF922897 |
| HC09 | - | - | AII | KF922977 | BIV | KF922898 |
| HC10 | A | JN116445 | AII | KF922978 | AII | KF922899 |
| HC11 | A | KF923021 | AII | KF922979 | AII | KF922900 |
| HC12 | A | JN116446 | AII | KF922980 | AII | KF922901 |
| HC13 | A | JN116447 | - | - | C | KF922902 |
| HC14 | - | - | - | - | BIV | KF922903 |
| HC15 | B | JN116448 | - | - | BIV | KF922904 |
| HC16 | B | JN116449 | - | - | BIV | KF922905 |
| HC17 | - | - | - | - | BIV | KF922906 |
| HC18 | A | JN116450 | - | - | - | - |
| HC19 | - | - | - | - | C | KF922907 |
| HC20 | - | - | - | - | BIV | KF922908 |
| HC21 | B | JN116451 | - | - | AII | KF922909 |
| HC22 | A | JN116452 | - | - | AII | KF922910 |
| HC23 | B | JN116453 | - | - | AII | KF922911 |
| HC24 | - | - | - | - | BIV | KF922912 |
| HC25 | B | JN116454 | B | KF922981 | BIV | KF922913 |
| HC26 | - | - | - | - | - | - |
| HC27 | A | JN116455 | A | KF922982 | AII | KF922914 |
| HC28 | A | JN116456 | - | - | - | - |
| HC29 | - | - | AII | KF922983 | AII | KF922915 |
| HC30 | B | JN116457 | - | - | BIV | KF922916 |
| HC31 | A | JN116458 | AII | KF922984 | AII | KF922917 |
| HC32 | B | JN116459 | BIV | KF922985 | BIV | KF922918 |
| HC33 | B | JN116460 | BIII | KF922986 | BIII | KF922919 |
| HC34 | B | JN116461 | B | KF922987 | BIV | KF922920 |
| HC35 | A | JN116462 | - | - | AII | KF922921 |
| HC36 | A | JN116463 | AII | KF922988 | AII | KF922922 |
| HC37 | - | - | - | - | BIV | KF922923 |
| HC38 | B | JN116464 | - | - | BIV | KF922924 |
| HC39 | B | JN116465 | BIV | KF922989 | BIV | KF922925 |
| HC40 | A | JN116466 | AII | KF922990 | AII | KF922926 |
| HC41 | B | JN116467 | - | - | BIV | KF922927 |
| HC42 | A | JN116468 | AII | KF922991 | AII | KF922928 |
| HC43 | B | JN116469 | - | - | BIV | KF922929 |
| HC44 | A | JN116470 | AII | KF922992 | AII | KF922930 |
| HC45 | B | JN116471 | BIV | KF922993 | BIV | KF922931 |
| HC46 | B | JN116472 | BIV | KF922994 | BIV | KF922932 |
| HC47 | B | JN116473 | BIV | KF922995 | BIV | KF922933 |
| HC48 | A | JN116474 | AII | KF922996 | AII | KF922934 |
| HC49 | A | JN116475 | AII | KF922997 | AII | KF922935 |
| HC50 | A | JN116476 | AII | KF922998 | AII | KF922936 |
| HC51 | A | JN116477 | AII | KF922999 | AII | KF922937 |
| HC43A* | - | - | - | - | BIV | KM495706 |
| HC43B* | - | - | - | - | BIV | KM495707 |
| HC43C* | - | - | - | - | BIV | KM495708 |

* Sequences obtained after molecular cloning.

**Table S3.** Molecular characterization and accession numbers of isolates obtained from day-care center based on sequencing data from *gdh*, *bg*, and *tpi* genes.

| **Sample** | ***gdh*** | ***GenBank*** | ***bg*** | ***GenBank*** | ***tpi*** | ***GenBank*** |
| --- | --- | --- | --- | --- | --- | --- |
| DC01 | B | JN116478 | AII | KF923000 | BIV | KF922938 |
| DC02 | - | - | AII | KF923001 | BIV | KF922939 |
| DC03 | B | JN116479 | AII | KF923002 | BIV | KF922940 |
| DC04 | B | JN116480 | - | - | BIII | KF922941 |
| DC05 | B | JN116481 | AII | KF923003 | BIV | KF922942 |
| DC06 | - | - | - | - | BIV | KF922943 |
| DC07 | A | JN116482 | AII | KF923004 | - | - |
| DC08 | - | - | AII | KF923005 | BIV | KF922944 |
| DC09 | B | JN116483 | - | - | BIII | KF922945 |
| DC10 | - | - | - | - | BIII | KF922946 |
| DC11 | - | - | AII | KF923006 | BIII | KF922947 |
| DC12 | A | JN116484 | AII | KF923007 | AII | KF922948 |
| DC13 | A | JN116485 | AII | KF923008 | - | - |
| DC14 | - | - | AII | KF923009 | BIV | KF922949 |
| DC15 | A | JN116486 | AII | KF923010 | AII | KF922950 |
| DC16 | B | JN116487 | - | - | BIV | KF922951 |
| DC17 | B | JN116488 | - | - | BIV | KF922952 |
| DC18 | - | - | - | - | BIII | KF922953 |
| DC19 | B | JN116489 | AII | KF923011 | BIV | KF922954 |
| DC20 | B | JN116490 | AII | KF923012 | BIV | KF922955 |
| DC21 | B | JN116491 | - | - | BIV | KF922956 |
| DC22 | B | JN116492 | AII | KF923013 | BIV | KF922957 |
| DC23 | - | - | AII | KF923014 | BIII | KF922958 |
| DC24 | - | - | - | - | BIV | KF922959 |
| DC25 | A | JN116493 | AII | KF923015 | AII | KF922960 |
| DC26 | - | - | A | KF923016 | - | - |
| DC27 | A | JN116494 | AII | KF923017 | AII | KF922961 |
| DC28 | A | JN116495 | AII | KF923018 | AII | KF922962 |

**Table S4.** Molecular characterization and accession numbers of isolates obtained from veterinary samples based on sequencing data from *gdh*, *bg*, and *tpi* genes.

| **Sample** | **Host** | ***gdh*** | ***GenBank*** | ***bg*** | ***GenBank*** | ***tpi*** | ***GenBank*** |
| --- | --- | --- | --- | --- | --- | --- | --- |
| VET01 | Dog | D | JN116498 | D | KF923019 | BIV | KF922963 |
| VET02 | Dog | D | JN116499 | - | - | AII | KF922964 |
| VET03 | Dog | C | JN116500 | - | - | - |  |
| VET04 | Cat | - | - | - | - | AII | KF922965 |
| VET05 | Cat | D | JN116497 | - | - | BIV | KF922966 |
| VET06 | Calf | E | JN116496 | E | KF923020 | AII | KF922967 |
| VET01L | Dog | - | - | - | - | D | KF922973 |
| VET02L | Dog | - | - | - | - | C | KF922974 |
| VET05L | Dog | - | - | - | - | D | KF922975 |
| VET02LA* | Dog | - | - | - | - | AI | KM495720 |
| VET02LB* | Dog | - | - | - | - | C | KM495721 |
| VET02LC* | Dog | - | - | - | - | C | KM495722 |
| VET04A* | Cat | - | - | - | - | BIII | KM495697 |
| VET04D* | Cat | - | - | - | - | BIV | KM495700 |
| VET04H* | Cat | - | - | - | - | C | KM495704 |

* Sequences obtained after molecular cloning.

**Table S5.** Molecular characterization and accession numbers of isolates obtained from environmental samples based on sequencing data from *gdh*, *bg*, and *tpi* genes.

| **Sample** | **Source** | ***gdh*** | ***GenBank*** | ***bg*** | ***GenBank*** | ***tpi*** | ***GenBank*** |
| --- | --- | --- | --- | --- | --- | --- | --- |
| ENV01 | SWWTP | A | JN116502 | - | - | - | - |
| ENV02 | Water Abstraction | - | - | - | - | C | KF922968 |
| ENV03 | Proença Stream | - | - | - | - | BIV | KF922969 |
| ENV04 | Serafim Stream | - | - | - | - | BIII | KF922970 |
| ENV05 | Anhumas River | D | JN116503 | - | - | BIV | KF922971 |
| ENV06 | Hospital Sewage | B | JN116504 | - | - | BIII | KF922972 |

**Table S6.** Distribution of each source as a percentage within each genetic assemblage.

| **Assemblage** | **A (%)** | **B (%)** | **C (%)** | **D (%)** | **E (%)** |
| --- | --- | --- | --- | --- | --- |
| HC | **52.1** | **46** | **62.5** | 0 | 0 |
| DC | 39.6 | 42 | 0 | 0 | 0 |
| VET | 6.2 | 4 | 25 | **75** | **100** |
| ENV | 2.1 | 8 | 12.5 | 25 | 0 |

Each genetic assemblage was examined to identify which source contributed with more isolates. Bold numbers indicate the highest percentage per column.

**Table S7.** Samples with double peaks within each loci.

| **Subtype** | **Nº of samples** | **Isolates** |
| --- | --- | --- |
| *gdh* | 22 | HC11 HC21 HC34 HC35 HC39 HC43 HC50 HC51 DC01 DC09 DC12 DC13 DC16 DC17 DC19 DC20 DC21 VET01 VET03 VET05 ENV01 ENV05 |
| *bg* | 11 | HC25 HC29 HC34 HC36 HC39 HC40 HC42 HC45 HC51 DC25 VET06 |
| *tpi* | 21 | HC14 HC15 HC20 HC21 HC23 HC24 HC25 HC40 HC42 HC43 HC45 HC50 HC51 DC06 DC25 VET01 VET04 VET05 VET06 ENV04 ENV05 |

**Table S8**. Correspondence between subtypes and sequences obtained from isolates from the *bg* gene*.*

| **Subtype** | **Marker** | **Isolates** |
| --- | --- | --- |
| **S01** | *bg* | HC44 DC02 DC03 DC05 DC07 DC11 DC12 DC13 DC14 DC15 DC19 DC22 DC23 DC27 DC28 |
| S02 | *bg* | HC10 HC11 HC49 |
| S03 | *bg* | HC31 HC48 HC50 |
| S04 | *bg* | HC46 HC47 |
| **S05** | *bg* | DC01 DC08 |
| S06 | *bg* | HC07 |
| **S07** | *bg* | HC09 |
| **S08** | *bg* | HC12 |
| **S09** | *bg* | HC27 |
| **S10** | *bg* | HC33 |
| **S11** | *bg* | DC20 |
| **S12** | *bg* | DC26 |
| **S13** | *bg* | VET01 |

Bold subtype numbers indicate new sequences.

**Table S9**. Correspondence between subtypes and sequences from isolates from the *gdh* gene*.*

| **Subtype** | **Marker** | **Isolates** |
| --- | --- | --- |
| S01 | *gdh* | HC01 HC02 HC10 HC12 HC13 HC18 HC22 HC27 HC28 HC31 HC36 HC40 HC42 HC44 HC48 HC49 DC07 DC15 DC25 DC27 DC28 |
| S02 | *gdh* | HC15 HC16 HC23 HC25 HC32 HC38 HC46 DC03 DC04 DC05 |
| S03 | *gdh* | HC45 DC22 ENV06 |
| S04 | *gdh* | HC41 HC47 |
| S05 | *gdh* | HC07 |
| **S06** | *gdh* | HC30 |
| S07 | *gdh* | HC33 |
| S08 | *gdh* | VET02 |
| **S09** | *gdh* | VET06 |

Bold subtype numbers indicate new sequences.

**Table S10**. Correspondence between subtypes and sequences obtained from the *tpi* gene*.*

| **Subtype** | **Marker** | **Isolates** |
| --- | --- | --- |
| S01 | *tpi* | HC09 HC16 HC32 HC34 HC37 HC41 HC46 HC47 DC03 DC05 DC14 DC16 DC17 DC19 DC21 DC22 DC24 |
| S02 | *tpi* | HC06 HC10 HC11 HC22 HC27 HC29 HC31 HC35 HC36 HC49 DC15 |
| **S03** | *tpi* | HC01 HC12 |
| S04 | *tpi* | HC33 DC18 |
| **S05** | *tpi* | HC44 DC27 |
| **S06** | *tpi* | DC01 DC08 |
| **S07** | *tpi* | DC04 DC10 |
| S08 | *tpi* | VET01L VET05L |
| **S09** | *tpi* | HC02 |
| **S10** | *tpi* | HC04 |
| S11 | *tpi* | HC07 |
| **S12** | *tpi* | HC08 |
| S13 | *tpi* | HC13 |
| **S14** | *tpi* | HC17 |
| **S15** | *tpi* | HC19 |
| S16 | *tpi* | HC30 |
| S17 | *tpi* | HC38 |
| **S18** | *tpi* | HC39 |
| **S19** | *tpi* | HC48 |
| **S20** | *tpi* | DC02 |
| **S21** | *tpi* | DC09 |
| S22 | *tpi* | DC11 |
| **S23** | *tpi* | DC12 |
| **S24** | *tpi* | DC20 |
| **S25** | *tpi* | DC23 |
| **S26** | *tpi* | DC28 |
| **S27** | *tpi* | VET02 |
| **S28** | *tpi* | VET02L |
| **S29** | *tpi* | ENV02 |
| **S30** | *tpi* | ENV03 |
| **S31** | *tpi* | ENV06 |

Bold subtype numbers indicate new sequences.

**Table S11.** Correspondence between haplotypes and sequences obtained from isolates from the *gdh* gene*.*

| **Haplotype** | **Molecular Marker** | **Assemblage** | **Isolates** |
| --- | --- | --- | --- |
| HP01 | *gdh* | AII | HC01 HC02 HC10 HC11 HC12 HC13 HC18 HC22 HC27 HC28 HC31 HC35 HC36 HC40 HC42 HC44 HC48 HC49 HC51 DC07 DC15 DC25 DC27 DC28 ENV01 |
| HP02 | *gdh* | BIV | HC15 HC16 HC23 HC25 HC32 HC38 HC46 DC03 DC04 DC05 |
| HP03 | *gdh* | BIV | HC39 HC45 DC22 ENV06 |
| HP04 | *gdh* | BIV | HC34 HC41 HC47 |
| HP05 | *gdh* | D | VET01 VET02 |
| HP06 | *gdh* | B | HC07 |
| HP07 | *gdh* | B | HC21 |
| HP08 | *gdh* | BIV | HC30 |
| HP09 | *gdh* | BIII | HC33 |
| HP10 | *gdh* | BIV | HC43 |
| HP11 | *gdh* | AII | HC50 |
| HP12 | *gdh* | B | DC01 |
| HP13 | *gdh* | BIII | DC09 |
| HP14 | *gdh* | AII | DC12 |
| HP15 | *gdh* | AII | DC13 |
| HP16 | *gdh* | BIV | DC16 |
| HP17 | *gdh* | BIV | DC17 |
| HP18 | *gdh* | BIV | DC19 |
| HP19 | *gdh* | BIV | DC20 |
| HP20 | *gdh* | BIV | DC21 |
| HP21 | *gdh* | C | VET03 |
| HP22 | *gdh* | D | VET05 |
| HP23 | *gdh* | E | VET06 |
| HP24 | *gdh* | D | ENV05 |

**Table S12.** Correspondence between haplotypes and sequences obtained from isolates from the *tpi* gene*.*

| **Haplotype** | | **Molecular Marker** | | **Assemblage** | | **Isolates** |
| --- | --- | --- | --- | --- | --- | --- |
| HP01 | *tpi* | | BIV | | HC09 HC14 HC15 HC16 HC20 HC32 HC34 HC37 HC41 HC45 HC46 HC47 HC43D DC03 DC05 DC14 DC16 DC17 DC19 DC21 DC22 DC24 ENV05 | |
| HP02 | *tpi* | | AII | | HC06 HC10 HC11 HC21 HC22 HC23 HC29 HC31 HC35 HC36 HC42 HC49 HC51 DC15 DC25 VET04 | |
| HP03 | *tpi* | | AII | | HC01 HC12 | |
| HP04 | *tpi* | | BIII | | HC33 DC18 | |
| HP05 | *tpi* | | AII | | HC44 DC27 | |
| HP06 | *tpi* | | BIV | | DC01 DC08 | |
| HP07 | *tpi* | | BIII | | DC04 DC10 | |
| HP08 | *tpi* | | D | | VET01L VET05L | |
| HP09 | *tpi* | | AII | | HC48 HC50 | |
| HP10 | *tpi* | | BIV | | HC43A HC43L | |
| HP11 | *tpi* | | BIV | | VET01 VET05 | |
| HP12 | *tpi* | | C | | HC02 | |
| HP13 | *tpi* | | C | | HC04 | |
| HP14 | *tpi* | | BIV | | HC07 | |
| HP15 | *tpi* | | C | | HC08 | |
| HP16 | *tpi* | | C | | HC13 | |
| HP17 | *tpi* | | BIV | | HC17 | |
| HP18 | *tpi* | | C | | HC19 | |
| HP19 | *tpi* | | BIV | | HC24 | |
| HP20 | *tpi* | | BIV | | HC25 | |
| HP21 | *tpi* | | AII | | HC27 | |
| HP22 | *tpi* | | BIV | | HC30 | |
| HP23 | *tpi* | | BIV | | HC38 | |
| HP24 | *tpi* | | BIV | | HC39 | |
| HP25 | *tpi* | | AII | | HC40 | |
| HP26 | *tpi* | | BIV | | HC43 | |
| HP27 | *tpi* | | BIV | | HC43B | |
| HP28 | *tpi* | | BIV | | HC43C | |
| HP29 | *tpi* | | BIV | | DC02 | |
| HP30 | *tpi* | | BIV | | DC06 | |
| HP31 | *tpi* | | AII | | DC12 | |
| HP32 | *tpi* | | BIV | | DC20 | |
| HP33 | *tpi* | | BIII | | DC09 | |
| HP34 | *tpi* | | BIII | | DC11 | |
| HP35 | *tpi* | | BIII | | DC23 | |
| HP36 | *tpi* | | AII | | DC28 | |
| HP37 | *tpi* | | AII | | VET02 | |
| HP38 | *tpi* | | AII | | VET06 | |
| HP39 | *tpi* | | C | | VET02L | |
| HP40 | *tpi* | | AI | | VET02LA | |
| HP41 | *tpi* | | C | | VET02LB | |
| HP42 | *tpi* | | C | | VET02LC | |
| HP43 | *tpi* | | BIII | | VET04A | |
| HP44 | *tpi* | | BIV | | VET04D | |
| HP45 | *tpi* | | C | | VET04H | |
| HP46 | *tpi* | | C | | ENV02 | |
| HP47 | *tpi* | | BIV | | ENV03 | |
| HP48 | *tpi* | | BIII | | ENV04 | |
| HP49 | *tpi* | | BIII | | ENV06 | |

**Table S13.** Correspondence between haplotypes and sequences obtained from isolates from the *bg* gene***.***

| **Haplotype** | | **Molecular Marker** | | **Assemblage** | | **Isolates** |
| --- | --- | --- | --- | --- | --- | --- |
| HP01 | *bg* | | AII | | DC02 DC03 DC05 DC07 DC11 DC12 DC13 DC14 DC15 DC19 DC22 DC23 DC27 | |
| HP02 | *bg* | | AII | | HC31 HC36 HC40 HC42 HC48 HC50 HC51 | |
| HP03 | *bg* | | AII | | HC10 HC11 HC44 HC49 DC25 | |
| HP04 | *bg* | | AII | | DC01 DC08 DC28 | |
| HP05 | *bg* | | BIV | | HC46 HC47 | |
| HP06 | *bg* | | BIII | | HC07 | |
| HP07 | *bg* | | AII | | HC09 | |
| HP08 | *bg* | | AII | | HC12 | |
| HP09 | *bg* | | BIV | | HC25 | |
| HP10 | *bg* | | AII | | HC27 | |
| HP11 | *bg* | | AII | | HC29 | |
| HP12 | *bg* | | BIV | | HC32 | |
| HP13 | *bg* | | BIII | | HC33 | |
| HP14 | *bg* | | BIV | | HC34 | |
| HP15 | *bg* | | BIV | | HC39 | |
| HP16 | *bg* | | BIV | | HC45 | |
| HP17 | *bg* | | AII | | DC20 | |
| HP18 | *bg* | | BIV | | DC26 | |
| HP19 | *bg* | | BIV | | VET01 | |
| HP20 | *bg* | | E | | VET06 | |

**Table S14.** Comparison of the main haplotype identified with sequences available at *GenBank* database.

| ***bg* haplotypes** | **n** | ***GenBank*** | ***tpi* haplotypes** | **n** | ***GenBank*** | ***gdh* haplotypes** | **n** | ***GenBank*** |
| --- | --- | --- | --- | --- | --- | --- | --- | --- |
| HP01 *bg* | 13 | - | HP01 *tpi* | 23 | JN587453  AB618783  HQ179644  HQ179645  HM140708  GU182385  FJ560559  EU518582  JN587453 | HP01 *gdh* | 25 | JX972184  JX448645  JN616252  KF468661  JN116441  JX266827 |
| HP02 *bg* | 7 | HQ179591  FJ971416  FN386484  AY072724 | HP02 *tpi* | 16 | GU564277  AB516351  FJ560552  JN587406  HQ603777  AB569403  GU182396 | HP02 *gdh* | 10 | KF468670  JX972186  KC313929  JX839875  JX448642  AB694737  JN204450  JQ700432 |

**Table S15.** Polymorphic sites in the *bg* and *tpi* sequences among *G. duodenalis* assemblage A isolates.

| **Genotype** | ***bg*** |  |  |  |  |  |  |  |  |  |  |  |
| --- | --- | --- | --- | --- | --- | --- | --- | --- | --- | --- | --- | --- |
|  | 40 | 52 | 76 | 165 | 167 | 173 | 251 | 421 | 429 | 549 | 567 | 621 |
| REF | A | C | A | A | A | A | A | C | T | C | C | C |
| BRA1 | - | - | - | - | - | - | . | . | . | . | T | . |
| BRA2 | . | . | . | . | . | . | . | T | C | . | T | T |
| BRA3 | . | . | . | . | . | . | . | T | C | . | T | T |
| BRA4 | - | - | - | - | - | - | . | T | C | . | T | T |
| BRA5 | C | A | . | . | . | . | . | T | C | . | T | . |
| BRA6 | G | A | C | C | C | C | . | . | . | . | G | . |
| BRA7 | - | - | - | - | - | - | G | . | . | T | - | - |
| **Genotype** | ***tpi*** |  |  |  |  |  |  |  |  |  |  |  |
|  |  |  | 42 | | 129 | | 349 | | 399 | |  | 510 |
| REF |  |  | T | | T | | G | | C | |  | G |
| BRA1 |  |  | . | | C | | . | | T | |  | . |
| BRA2 |  |  | . | | C | | . | | T | |  | . |
| BRA3 |  |  | . | | C | | . | | T | |  | A |
| BRA4 |  |  | C | | C | | . | | T | |  | . |
| BRA5 |  |  | . | | C | | . | | T | |  | . |
| BRA6 |  |  | . | | C | | A | | T | |  | . |
| BRA7 |  |  | - | | C | | . | | T | |  | . |

The *gdh* sequences were not inserted as they did not present any polymorphic site among the seven multilocus genotypes. Dots indicate identity to the reference sequence. Nucleotide substitutions are numbered from the ATG codon of each gene.

**Table S16.** Polymorphic sites in the *bg*, *tpi* and *gdh* sequences among *G. duodenalis* assemblage A isolates.

| **Genotype** | ***bg*** | | | | | | | | | | | | | |
| --- | --- | --- | --- | --- | --- | --- | --- | --- | --- | --- | --- | --- | --- | --- |
|  |  |  | 40 | 46 | 52 | 79 | 165 | 171 | 189 | 288 | 330 | 399 | 477 | 609 |
| Reference |  |  | A | G | C | A | G | T | A | T | C | T | T | A |
| BRAB1 |  |  | G | C | A | C | . | C | G | C | . | C | . | G |
| BRAB2 |  |  | . | . | . | . | A | . | . | . | T | . | C | G |
| BRAB3 |  |  | . | . | . | . | A | . | . | . | T | . | C | G |
|  |  |  |  |  |  |  |  |  |  |  |  |  |  |  |
| **Genotype** | ***tpi*** | | | | | | | | | | | | | |
|  | 39 | | 91 | | 162 | | 165 | | 168 | | 210 | | 429 | |
| Reference | A | | T | | G | | T | | T | | A | | G | |
| BRAB1 | G | | C | | A | | C | | C | | G | | . | |
| BRAB2 | . | | . | | . | | . | | . | | . | | A | |
| BRAB3 | . | | . | | . | | . | | . | | . | | A | |
|  |  |  |  |  |  |  |  |  |  |  |  |  |  |  |
| **Genotype** | ***gdh*** | | | | | | | | | | | | | |
|  | 309 | | | 354 | | |  |  |  |  |  |  |  |  |
| Reference | T | | | G | | |  |  |  |  |  |  |  |  |
| BRAB1 | C | | | . | | |  |  |  |  |  |  |  |  |
| BRAB2 | . | | | . | | |  |  |  |  |  |  |  |  |
| BRAB3 | . | | | A | | |  |  |  |  |  |  |  |  |

Dots indicate identity to the reference sequence. Nucleotide substitutions are numbered from the ATG codon of each gene.

**Table S17.** Multilocus genotypes (MLGs) in *G. duodenalis* assemblages A isolates.

| **MLG** | **Samples** | ***bg*** | ***gdh*** | ***tpi*** | **Assemblage** |
| --- | --- | --- | --- | --- | --- |
| BRA1 | HC31/HC48* | BG-1 (kc8) | gdh-1 | tpi-1 (Ad2) | AII |
| BRA2 | DC15/**DC07** | BG-2 | gdh-1 | - | AII |
| BRA3 | DC27/HC44 | BG-2 | gdh-1 | tpi-2 | AII |
| BRA4 | DC28 | BG-2 | gdh-1 | tpi-3 | AII |
| BRA5 | HC10/HC49* | BG-3 | gdh-1 | tpi-1 (Ad2) | AII |
| BRA6 | HC12/**HC01** | - | gdh-1 | tpi-4 | AII |
| BRA7 | HC27* | BG-5 | gdh-1 | tpi-1 (Ad2) | AII |

* MLGs where HC22 isolate could be included as data from *bg* gene was not obtained

**Table S18.** Multilocus genotypes (MLGs) in *G. duodenalis* assemblages B isolates.

| **MLG** | **Sample** | ***bg*** | ***gdh*** | ***tpi*** | **Assemblage** |
| --- | --- | --- | --- | --- | --- |
| BRAB1 | HC33 | BGB-1 | gdhB-2 | tpiB-2 | BIV |
| BRAB2 | HC46/**HC16 HC41** | - | gdhB-1 (Ad7) | tpiB-1 (Ad19) | BIV |
| BRAB3 | HC47 | BGB-2 | gdhB-3 | tpiB-1 (Ad19) | BIV |
| BRAB4 | **DC04** | - | gdhB-1 (Ad7) | tpiB-3 | BIII |
| BRAB5 | **ENV06** | - | gdhB-4 | tpiB-4 | BIII |
| BRAB6 | **HC38** | - | gdhB-1 (Ad7) | tpiB-5 | BIV |
| BRAB7 | **HC30** | - | gdhB-5 | tpiB-6 | BIV |

**Table S19.** Genetic differences in the genetic assemblages at the *gdh* locus.

|  | **Site number** | | | |  |  |  |  |  |  |  |  |  |  |  |  |  |  |  |  |  |  |  |  |  |  |  |  |  |  |  |  |  |  |  |
| --- | --- | --- | --- | --- | --- | --- | --- | --- | --- | --- | --- | --- | --- | --- | --- | --- | --- | --- | --- | --- | --- | --- | --- | --- | --- | --- | --- | --- | --- | --- | --- | --- | --- | --- | --- |
| ***gdh*** |  | | | **1** | **1** | **2** | **3** | **3** | **4** | **4** | **4** | **5** | **5** | **6** | **6** | **7** | **7** | **8** | **8** | **9** | **9** | **0** | **1** | **1** | **2** | **2** | **3** | **3** | **4** | **5** | **6** | **6** | **6** | **7** | **7** |
|  | **1** | **4** | **7** | **6** | **9** | **2** | **1** | **4** | **0** | **3** | **6** | **5** | **8** | **4** | **7** | **0** | **6** | **2** | **8** | **1** | **4** | **6** | **2** | **8** | **4** | **7** | **3** | **9** | **5** | **1** | **3** | **6** | **9** | **2** | **5** |
| **A L40509.1** | G | T | C | C | T | C | C | G | C | C | C | C | G | C | C | C | C | C | T | C | A | C | C | C | C | C | G | C | C | C | C | C | C | C | T |
| **BIII AF069059.1** | . | C | . | . | C | T | . | . | . | . | T | T | . | . | T | T | . | . | C | T | T | G | . | . | . | T | . | T | T | . | . | G | T | . | C |
| **BIV L40508** | . | C | T | . | C | T | . | . | . | . | T | T | . | . | T | T | . | . | C | T | T | G | . | . | . | . | . | T | . | . | . | G | T | . | C |
| **C U60985** | A | C | . | T | C | . | . | T | . | . | . | . | C | . | T | . | T | . | C | . | C | . | . | . | . | . | . | . | . | . | . | . | T | . | C |
| **D U60986.2** | . | C | T | T | C | T | T | A | T | T | . | . | C | . | T | . | T | T | C | . | C | T | T | T | . | . | A | T | . | T | . | . | T | . | . |
| **E AY178740** | A | C | . | . | C | . | T | . | . | . | . | . | . | . | T | . | . | . | . | . | G | T | . | . | T | . | . | . | T | T | T | G | T | . | . |
| **F AY178744** | C | C | . | . | C | . | . | . | . | . | . | . | . | . | . | . | . | . | . | . | G | . | . | . | . | . | . | . | . | . | . | . | . | T | C |
| **G AY178748** | . | C | . | . | C | . | . | . | . | . | . | . | . | T | T | . | T | . | C | . | G | A | . | . | . | . | . | . | . | . | . | G | T | . | C |

All the SNPs in the different genetic assemblages are displayed within *gdh* loci.

**Table S20.** Genetic differences in the genetic assemblages at the *bg* locus.

| ***bg*** |  |  | **Site numbers** | | | |  |  | **1** | **1** | **1** | **1** | **1** | **1** | **1** | **1** | **1** | **1** | **1** | **1** | **1** | **1** | **1** | **1** | **1** | **2** | **2** | **2** | **2** | **2** | **2** | **2** | **2** | **2** | **2** | **2** | **2** | **2** | **2** |
| --- | --- | --- | --- | --- | --- | --- | --- | --- | --- | --- | --- | --- | --- | --- | --- | --- | --- | --- | --- | --- | --- | --- | --- | --- | --- | --- | --- | --- | --- | --- | --- | --- | --- | --- | --- | --- | --- | --- | --- |
|  |  |  | **1** | **1** | **2** | **5** | **6** | **9** | **0** | **0** | **0** | **2** | **2** | **3** | **3** | **3** | **4** | **5** | **5** | **7** | **7** | **7** | **7** | **8** | **9** | **0** | **0** | **1** | **1** | **2** | **2** | **2** | **3** | **3** | **3** | **4** | **4** | **5** | **5** |
|  |  | **4** | **0** | **3** | **2** | **5** | **1** | **7** | **3** | **6** | **9** | **4** | **7** | **0** | **3** | **9** | **5** | **1** | **7** | **5** | **6** | **7** | **8** | **1** | **9** | **2** | **5** | **4** | **7** | **0** | **3** | **9** | **0** | **2** | **5** | **4** | **7** | **0** | **3** |
| **AI \|M36728** |  | C | G | G | C | C | G | C | G | C | G | C | C | C | A | C | G | C | G | G | G | C | C | G | G | G | C | C | C | C | C | C | C | A | G | G | G | C | C |
| **AII \|AY072723** |  | . | . | . | . | . | . | . | . | . | . | . | . | . | . | . | . | . | . | . | . | . | . | . | . | . | . | . | . | . | . | . | . | . | . | . | . | . | . |
| **AIII DQ648777** |  | . | . | . | . | . | . | . | . | . | . | . | . | . | . | . | . | . | . | . | . | . | . | . | . | . | . | . | . | . | T | . | . | . | . | . | . | . | . |
| **BIII \|AY072726** |  | T | . | A | . | . | . | . | . | T | . | . | . | . | G | . | A | . | A | . | . | . | . | . | . | . | . | T | . | . | . | . | . | G | . | . | . | . | . |
| **BIV \|AY072725** |  | T | . | A | . | . | . | . | . | T | . | . | . | . | G | T | A | . | A | . | . | . | . | . | . | A | . | T | . | . | . | . | . | G | . | . | . | . | . |
| **C \|JF422719** |  | - | - | - | - | - | - | . | . | . | C | . | . | . | T | G | . | . | C | . | . | G | . | . | . | . | . | . | . | . | . | . | . | G | . | . | A | . | . |
| **D \|AY545647** |  | T | A | A | T | T | . | T | . | T | C | T | . | . | . | T | . | T | . | A | A | G | . | C | . | . | A | . | T | . | . | T | . | G | . | . | . | T | . |
| **E \|AY072729** |  | . | . | . | . | . | A | . | . | . | . | . | . | T | . | . | . | . | . | . | . | . | . | . | . | . | . | T | . | T | . | . | . | G | A | A | . | . | . |
| **F \|AY647264** |  | . | . | . | . | . | . | . | . | . | . | . | . | . | G | . | . | . | . | . | . | . | . | . | . | . | . | . | . | . | . | . | . | G | . | . | . | . | . |
| **G \|EU769221** |  | - | - | - | - | - | - | T | A | . | . | T | G | . | . | T | . | . | . | . | . | . | G | . | A | . | . | . | . | . | . | . | T | G | . | . | . | . | T |
|  | **2** | **2** | **2** | **2** | **2** | **2** | **2** | **2** | **3** | **3** | **3** | **3** | **3** | **3** | **3** | **3** | **3** | **3** | **3** | **3** | **3** | **4** | **4** | **4** | **4** | **4** | **4** | **4** | **4** | **4** | **4** | **4** | **4** | **4** | **4** | **4** | **5** | **5** | **5** |
|  | **5** | **6** | **6** | **8** | **8** | **8** | **8** | **9** | **0** | **1** | **1** | **1** | **2** | **4** | **5** | **6** | **6** | **6** | **7** | **7** | **9** | **0** | **0** | **0** | **1** | **1** | **2** | **3** | **4** | **6** | **7** | **7** | **8** | **8** | **9** | **9** | **0** | **1** | **2** |
|  | **6** | **2** | **5** | **0** | **3** | **6** | **9** | **8** | **1** | **0** | **3** | **9** | **2** | **6** | **2** | **1** | **4** | **7** | **0** | **9** | **7** | **0** | **6** | **9** | **2** | **5** | **7** | **0** | **5** | **3** | **5** | **8** | **4** | **7** | **3** | **6** | **2** | **7** | **6** |
| **AI \|M36728** | C | T | A | C | T | G | G | C | A | C | T | A | T | T | C | G | T | C | G | C | T | C | G | C | T | C | A | A | C | G | C | C | C | C | C | G | G | C | G |
| **AII \|AY072723** | . | . | . | . | . | . | . | . | . | . | . | . | . | . | . | . | . | . | . | . | . | . | . | . | . | . | . | . | . | . | . | . | . | . | . | . | . | . | . |
| **AIII DQ648777** | . | . | . | . | . | . | . | . | . | . | . | . | C | C | . | . | . | . | . | . | C | . | . | . | C | . | . | G | . | . | . | . | . | . | . | . | A | . | . |
| **BIII \|AY072726** | . | C | . | T | C | C | . | . | G | . | C | G | C | C | . | A | C | . | . | . | C | . | A | . | C | . | C | G | T | A | . | . | . | . | T | C | . | . | . |
| **BIV \|AY072725** | T | C | . | T | . | T | . | . | G | . | C | G | C | C | . | A | C | T | . | . | C | . | A | . | C | . | C | G | T | A | . | . | . | . | T | C | . | . | . |
| **C \|JF422719** | . | C | G | . | . | C | . | G | C | . | C | . | C | C | . | . | C | . | . | . | C | . | C | . | C | . | C | G | . | . | A | . | . | . | . | C | . | . | . |
| **D \|AY545647** | A | C | . | . | C | A | . | G | G | . | C | . | C | C | . | A | . | . | . | . | C | T | C | . | C | T | C | G | . | . | A | . | T | T | T | C | . | . | A |
| **E \|AY072729** | . | . | . | T | C | . | A | . | . | . | . | . | C | C | T | . | C | . | A | . | C | . | . | . | C | . | . | G | T | A | . | T | . | . | . | C | . | T | . |
| **F \|AY647264** | . | A | C | . | . | A | . | . | . | T | . | . | C | C | . | . | C | . | . | . | C | . | . | . | C | G | . | G | . | . | . | . | . | . | . | C | . | . | . |
| **G \|EU769221** | . | C | G | . | C | . | . | . | G | . | . | . | C | C | . | . | C | . | . | A | C | . | . | A | . | T | C | G | . | . | . | . | . | . | . | C | . | T | . |
|  |  |  |  |  |  |  |  |  |  |  |  |  |  |  |  |  |  |  |  |  |  |  |  |  |  |  |  |  |  |  |  |  |  |  |  |  |  |  |  |
|  | **5** | **5** | **5** | **5** | **5** | **5** | **5** | **5** | **5** | **5** | **5** | **6** | **6** | **6** | **6** | **6** | **6** | **6** | **6** | **6** | **6** | **6** | **6** | **6** |  |  |  |  |  |  |  |  |  |  |  |  |  |  |  |
|  | **3** | **3** | **3** | **4** | **4** | **5** | **5** | **7** | **7** | **8** | **9** | **0** | **1** | **2** | **2** | **2** | **3** | **4** | **5** | **5** | **7** | **7** | **8** | **9** |  |  |  |  |  |  |  |  |  |  |  |  |  |  |  |
|  | **2** | **5** | **8** | **4** | **7** | **0** | **3** | **1** | **7** | **0** | **5** | **7** | **9** | **2** | **5** | **8** | **1** | **3** | **5** | **8** | **0** | **6** | **5** | **1** |  |  |  |  |  |  |  |  |  |  |  |  |  |  |  |
| **AI \|M36728** | T | C | T | G | A | A | G | C | G | G | G | C | C | C | T | C | G | C | C | A | A | C | C | T |  |  |  |  |  |  |  |  |  |  |  |  |  |  |  |
| **AII \|AY072723** | . | T | . | . | . | . | . | . | . | . | . | . | . | . | . | . | . | . | . | G | . | . | . | . |  |  |  |  |  |  |  |  |  |  |  |  |  |  |  |
| **AIII DQ648777** | C | . | . | . | . | G | . | . | . | . | . | . | . | . | C | . | . | . | . | G | G | T | . | . |  |  |  |  |  |  |  |  |  |  |  |  |  |  |  |
| **BIII \|AY072726** | C | . | C | . | . | G | C | . | . | . | . | . | . | . | C | . | . | . | A | G | . | . | . | A |  |  |  |  |  |  |  |  |  |  |  |  |  |  |  |
| **BIV \|AY072725** | C | . | C | . | . | G | C | . | A | . | . | . | . | . | C | . | . | . | A | G | . | . | . | A |  |  |  |  |  |  |  |  |  |  |  |  |  |  |  |
| **C \|JF422719** | C | . | A | . | C | G | . | T | . | A | A | . | . | . | C | . | . | T | . | G | G | . | T | G |  |  |  |  |  |  |  |  |  |  |  |  |  |  |  |
| **D \|AY545647** | C | . | A | A | . | G | T | . | . | . | . | T | . | T | C | . | A | . | A | G | . | . | . | C |  |  |  |  |  |  |  |  |  |  |  |  |  |  |  |
| **E \|AY072729** | C | . | C | . | G | G | C | . | . | . | . | . | T | . | C | T | . | . | . | G | . | . | . | . |  |  |  |  |  |  |  |  |  |  |  |  |  |  |  |
| **F \|AY647264** | C | . | C | . | . | G | . | . | . | . | . | . | . | . | C | . | . | . | . | G | G | . | . | G |  |  |  |  |  |  |  |  |  |  |  |  |  |  |  |
| **G \|EU769221** | C | . | C | A | G | G | . | - | - | - | - | - | - | - | - | - | - | - | - | - | - | - | - | - |  |  |  |  |  |  |  |  |  |  |  |  |  |  |  |

All the SNPs in the different genetic assemblages are displayed within *bg* loci.

**Table S21.** Genetic differences in the genetic assemblages at the *tpi* locus.

| ***tpi*** |  | **Site number** | | | |  |  |  |  |  |  |  |  |  |  |  |  |  |  |  |  |  |  |  |  |  |  |  |  |  |  |  |  |  |  |  |  |  |  |
| --- | --- | --- | --- | --- | --- | --- | --- | --- | --- | --- | --- | --- | --- | --- | --- | --- | --- | --- | --- | --- | --- | --- | --- | --- | --- | --- | --- | --- | --- | --- | --- | --- | --- | --- | --- | --- | --- | --- | --- |
|  |  |  |  |  | **1** | **1** | **1** | **1** | **2** | **2** | **2** | **2** | **3** | **3** | **3** | **4** | **4** | **4** | **4** | **4** | **4** | **4** | **5** | **5** | **5** | **5** | **6** | **6** | **6** | **7** | **7** | **7** | **7** | **8** | **8** | **8** | **8** | **8** |  |
|  |  | **1** | **4** | **7** | **0** | **3** | **6** | **9** | **1** | **2** | **5** | **8** | **1** | **4** | **7** | **0** | **1** | **2** | **3** | **4** | **6** | **9** | **2** | **3** | **6** | **8** | **1** | **4** | **7** | **0** | **3** | **6** | **9** | **2** | **3** | **5** | **6** | **8** |  |
| **AI \|L02120.1** |  | C | G | T | C | C | T | T | A | C | T | C | G | C | C | G | G | C | G | G | A | T | T | G | C | T | G | C | T | T | C | G | C | C | G | C | A | T |  |
| **AII \|U57897** |  | . | . | . | . | . | . | . | . | . | . | . | . | . | . | . | . | . | . | . | . | . | . | . | . | . | . | . | . | . | . | . | . | . | . | . | . | . |  |
| **AIII \|DQ650648** |  | . | . | . | . | . | . | . | . | . | . | . | . | . | . | . | . | . | . | . | . | . | . | . | . | C | . | . | . | . | T | A | . | T | . | T | . | . |  |
| **BIII \|AY228628** |  | . | . | C | T | A | G | C | . | . | C | T | . | . | . | A | . | . | . | T | C | C | C | T | . | . | . | . | C | G | . | . | . | T | . | T | G | . |  |
| **BIV \|L02116** |  | . | A | C | T | A | G | C | . | . | C | T | . | . | . | A | . | . | . | T | C | C | C | T | T | . | . | . | C | G | . | . | . | T | . | T | G | . |  |
| **C \|AY228641** |  | T | . | C | . | G | G | . | . | . | . | . | A | . | T | A | . | . | . | . | C | C | G | T | . | C | . | T | C | C | T | T | T | G | A | . | . | C |  |
| **D \|DQ246216** |  | T | . | C | . | G | . | . | C | . | . | . | . | T | T | . | T | . | T | . | T | . | . | T | . | C | C | . | C | . | T | . | T | G | A | T | . | . |  |
| **E \|AY655705** |  | T | . | . | . | . | G | . | . | T | . | . | . | . | T | . | . | . | . | . | C | . | . | . | . | . | . | . | C | . | . | A | . | T | . | T | G | . |  |
| **F \|AF069558** |  | T | . | C | . | . | G | C | . | . | . | . | . | T | . | . | . | G | . | . | C | . | . | . | . | C | . | . | . | . | . | . | . | T | . | T | G | C |  |
| **G \|EU781013** |  | T | . | C | . | A | G | C | . | . | C | . | . | . | . | . | . | . | A | . | C | A | . | T | . | C | . | . | C | . | T | . | . | T | . | G | . | . |  |
|  |  |  |  |  |  |  |  |  |  |  |  |  |  |  |  |  |  |  |  |  |  |  |  |  |  |  |  |  |  |  |  |  |  |  |  |  |  |  |  |
|  |  |  |  |  |  | **1** | **1** | **1** | **1** | **1** | **1** | **1** | **1** | **1** | **1** | **1** | **1** | **1** | **1** | **1** | **1** | **1** | **1** | **1** | **1** | **1** | **1** | **1** | **1** | **1** | **1** | **1** | **1** | **1** | **1** | **1** | **1** | **1** | **1** |
|  | **9** | **9** | **9** | **9** | **9** | **0** | **0** | **0** | **0** | **0** | **1** | **1** | **1** | **2** | **2** | **2** | **3** | **3** | **3** | **3** | **4** | **4** | **4** | **4** | **4** | **4** | **5** | **5** | **6** | **6** | **6** | **7** | **7** | **7** | **7** | **8** | **8** | **8** | **9** |
|  | **1** | **4** | **7** | **8** | **9** | **0** | **3** | **6** | **7** | **9** | **2** | **5** | **8** | **1** | **4** | **7** | **0** | **3** | **6** | **9** | **0** | **1** | **2** | **3** | **7** | **8** | **4** | **7** | **0** | **3** | **9** | **0** | **2** | **5** | **8** | **1** | **4** | **7** | **3** |
| **AI \|L02120.1** | C | T | C | G | C | C | A | C | C | G | A | A | C | T | G | A | C | G | A | A | C | A | G | T | G | G | A | G | G | T | C | C | A | G | G | C | G | G | T |
| **AII \|U57897** | . | C | . | . | . | . | . | . | . | . | . | . | . | . | . | . | . | . | . | . | . | . | . | . | . | . | . | . | . | . | . | . | . | . | . | . | . | . | . |
| **AIII \|DQ650648** | . | . | . | T | . | . | . | . | . | A | . | . | . | . | . | G | . | . | . | G | . | . | . | . | . | . | G | . | . | . | . | . | . | . | . | . | . | . | . |
| **BIII \|AY228628** | T | C | . | T | T | T | G | . | . | T | T | . | T | . | . | G | . | C | G | G | T | G | T | C | A | A | . | A | . | C | T | . | G | . | . | . | T | A | C |
| **BIV \|L02116** | T | C | . | T | T | T | G | . | . | T | T | . | T | . | . | G | T | T | G | G | T | G | T | C | A | A | . | A | . | C | T | . | G | A | . | . | T | A | C |
| **C \|AY228641** | . | C | G | T | . | . | G | T | . | . | T | G | . | C | A | G | . | A | G | G | . | . | . | C | A | . | . | . | . | . | . | . | C | . | A | . | C | A | G |
| **D \|DQ246216** | T | . | G | T | . | G | G | T | . | A | T | G | T | . | A | C | T | . | . | G | . | . | . | C | A | . | . | . | A | . | . | T | C | A | T | T | C | . | . |
| **E \|AY655705** | . | . | T | . | . | . | . | T | T | A | . | . | T | . | A | . | . | . | . | . | . | . | . | . | A | . | G | . | . | . | . | . | . | . | . | T | A | . | . |
| **F \|AF069558** | . | . | T | . | . | . | G | . | . | . | . | . | . | . | . | . | . | . | . | G | . | . | . | . | A | . | G | . | . | . | . | . | . | . | . | . | . | . | C |
| **G \|EU781013** | . | C | . | . | . | T | G | . | . | . | T | G | . | C | . | . | . | . | G | G | . | . | . | C | A | . | G | . | . | . | . | . | G | . | C | . | C | . | G |
|  | **1** | **1** | **2** | **2** | **2** | **2** | **2** | **2** | **2** | **2** | **2** | **2** | **2** | **2** | **2** | **2** | **2** | **2** | **2** | **2** | **2** | **2** | **2** | **2** | **2** | **2** | **2** | **2** | **2** | **2** | **2** | **2** | **2** | **2** | **3** | **3** | **3** | **3** | **3** |
|  | **9** | **9** | **0** | **0** | **0** | **1** | **1** | **2** | **2** | **2** | **2** | **3** | **3** | **3** | **3** | **3** | **3** | **3** | **4** | **4** | **4** | **5** | **5** | **6** | **6** | **6** | **7** | **8** | **8** | **8** | **8** | **9** | **9** | **9** | **0** | **0** | **1** | **1** | **1** |
|  | **6** | **9** | **2** | **5** | **8** | **7** | **9** | **0** | **3** | **8** | **9** | **0** | **2** | **3** | **4** | **5** | **6** | **8** | **1** | **5** | **7** | **0** | **3** | **2** | **5** | **8** | **7** | **0** | **3** | **4** | **6** | **2** | **5** | **8** | **0** | **7** | **3** | **6** | **7** |
| **AI \|L02120.1** | C | G | A | T | T | T | A | G | C | G | T | T | G | A | A | G | C | T | G | G | A | G | C | A | C | A | G | G | C | G | C | A | C | C | A | T | T | C | C |
| **AII \|U57897** | . | . | . | . | . | . | . | . | . | . | . | . | . | . | . | . | . | . | . | . | . | . | . | . | . | . | . | . | . | . | . | . | . | . | . | . | . | . | . |
| **AIII \|DQ650648** | T | . | . | . | . | . | . | . | . | . | . | . | . | . | . | . | . | . | . | . | . | . | . | . | . | . | . | . | . | . | . | . | . | . | . | . | . | . | T |
| **BIII \|AY228628** | . | . | . | C | C | G | T | . | . | . | G | C | . | . | G | C | . | . | A | A | . | A | . | . | T | . | C | . | . | A | T | G | T | T | . | G | . | T | . |
| **BIV \|L02116** | . | . | . | C | C | G | T | . | . | . | G | C | . | . | G | C | . | . | A | A | . | A | . | . | T | . | C | . | . | A | T | G | T | T | . | G | . | T | . |
| **C \|AY228641** | . | . | . | . | . | . | . | . | . | . | C | C | . | . | G | T | . | C | . | . | . | . | . | . | T | G | C | . | . | A | . | G | T | . | . | . | . | T | . |
| **D \|DQ246216** | G | A | . | C | . | A | T | T | T | A | C | . | A | . | G | T | . | . | . | A | T | . | . | . | . | . | C | A | T | A | T | . | . | . | . | C | . | T | T |
| **E \|AY655705** | T | . | G | . | . | . | . | . | . | . | C | . | . | G | . | . | T | C | . | A | . | . | T | G | T | . | . | . | . | . | . | G | T | . | . | . | . | T | . |
| **F \|AF069558** | T | . | G | C | . | C | . | . | . | . | C | C | A | G | . | . | . | . | . | A | . | . | . | G | . | . | . | . | . | . | . | G | . | . | G | G | C | T | . |
| **G \|EU781013** | . | A | . | C | C | C | T | . | . | . | C | C | . | . | G | C | . | C | . | . | T | . | . | . | . | G | C | . | . | . | . | G | . | G | . | C | . | . | . |
|  |  |  |  |  |  |  |  |  |  |  |  |  |  |  |  |  |  |  |  |  |  |  |  |  |  |  |  |  |  |  |  |  |  |  |  |  |  |  |  |
|  |  |  |  |  |  |  |  |  |  |  |  |  |  |  |  |  |  |  |  |  |  |  |  |  |  |  |  |  |  |  |  |  |  |  |  |  |  |  |  |
|  | **3** | **3** | **3** | **3** | **3** | **3** | **3** | **3** | **3** | **3** | **3** | **3** | **3** | **3** | **3** | **3** | **3** | **3** | **3** | **3** | **3** | **3** | **3** | **3** | **3** | **3** | **3** | **3** | **3** | **3** | **3** | **4** | **4** | **4** | **4** | **4** | **4** | **4** | **4** |
|  | **1** | **2** | **2** | **2** | **2** | **3** | **3** | **3** | **4** | **4** | **4** | **4** | **4** | **4** | **5** | **5** | **5** | **5** | **6** | **6** | **6** | **7** | **7** | **8** | **8** | **8** | **8** | **9** | **9** | **9** | **9** | **0** | **0** | **0** | **0** | **0** | **1** | **1** | **1** |
|  | **9** | **2** | **5** | **7** | **8** | **3** | **4** | **7** | **0** | **3** | **6** | **7** | **8** | **9** | **2** | **5** | **8** | **9** | **1** | **4** | **7** | **0** | **9** | **0** | **1** | **2** | **5** | **0** | **1** | **4** | **7** | **0** | **2** | **3** | **6** | **9** | **5** | **8** | **9** |
| **AI \|L02120.1** | G | A | G | G | G | C | G | C | C | C | C | G | T | C | A | G | C | T | G | C | G | C | C | C | G | C | C | A | G | G | C | C | C | C | G | T | G | T | G |
| **AII \|U57897** | . | . | . | . | . | . | . | . | . | . | . | . | . | . | . | . | . | . | . | T | . | . | . | . | . | . | . | . | . | . | . | . | . | . | . | . | . | . | . |
| **AIII \|DQ650648** | . | . | . | . | . | . | . | . | . | . | . | . | . | . | . | . | . | C | . | . | . | . | . | . | . | . | . | . | . | . | . | . | . | . | . | . | . | . | . |
| **BIII \|AY228628** | . | C | A | . | T | . | T | T | . | . | . | A | C | . | . | . | . | C | . | T | A | . | T | A | A | . | T | . | . | . | T | T | . | T | . | C | T | . | A |
| **BIV \|L02116** | . | C | A | . | T | . | T | T | . | . | . | A | C | . | . | . | . | C | . | T | A | . | T | A | A | . | T | . | . | . | T | T | . | T | . | C | T | . | A |
| **C \|AY228641** | . | G | . | . | C | T | . | . | . | . | . | A | C | T | G | . | A | C | . | . | . | . | . | A | A | G | T | . | T | . | . | T | G | A | . | C | C | . | A |
| **D \|DQ246216** | A | G | A | . | T | T | T | . | T | . | T | A | . | A | . | A | T | C | . | T | A | . | T | A | A | G | . | . | T | . | T | T | . | G | . | . | C | A | A |
| **E \|AY655705** | A | . | . | A | T | . | . | T | . | T | T | . | . | T | . | . | . | C | T | T | . | . | . | . | . | . | . | . | . | A | . | T | . | T | . | . | . | C | A |
| **F \|AF069558** | A | . | . | . | . | . | . | . | . | . | . | . | . | A | . | . | . | C | . | . | . | . | . | . | . | . | . | . | . | . | . | . | . | . | . | . | . | C | A |
| **G \|EU781013** | . | T | . | . | C | . | C | . | . | . | T | A | . | . | . | . | . | C | A | T | . | T | . | A | A | . | . | C | . | . | . | T | G | . | A | C | C | C | A |
|  |  |  |  |  |  |  |  |  |  |  |  |  |  |  |  |  |  |  |  |  |  |  |  |  |  |  |  |  |  |  |  |  |  |  |  |  |  |  |  |
|  | **4** | **4** | **4** | **4** | **4** | **4** | **4** | **4** | **4** | **4** | **4** | **4** | **4** | **4** | **4** | **4** | **4** | **4** | **4** | **4** | **4** | **4** | **4** | **4** | **4** | **4** | **4** | **4** | **4** | **4** |  |  |  |  |  |  |  |  |  |
|  | **2** | **2** | **2** | **2** | **2** | **3** | **3** | **3** | **3** | **3** | **4** | **4** | **4** | **4** | **5** | **5** | **5** | **5** | **5** | **6** | **6** | **6** | **6** | **6** | **7** | **7** | **7** | **8** | **8** | **9** |  |  |  |  |  |  |  |  |  |
|  | **0** | **1** | **4** | **7** | **8** | **0** | **3** | **6** | **7** | **9** | **3** | **4** | **6** | **8** | **2** | **4** | **5** | **6** | **7** | **0** | **1** | **3** | **6** | **9** | **2** | **5** | **8** | **1** | **7** | **0** |  |  |  |  |  |  |  |  |  |
| **AI \|L02120.1** | G | C | G | G | C | C | A | G | T | C | A | T | C | C | A | G | G | A | G | T | G | C | T | T | C | G | C | G | C | T |  |  |  |  |  |  |  |  |  |
| **AII \|U57897** | . | . | . | . | . | . | . | . | . | . | . | . | . | . | . | . | . | . | . | . | . | . | . | . | . | . | . | . | . | . |  |  |  |  |  |  |  |  |  |
| **AIII \|DQ650648** | . | . | . | . | . | . | . | . | . | . | . | . | . | . | . | . | . | . | T | . | . | T | . | . | . | . | . | . | . | . |  |  |  |  |  |  |  |  |  |
| **BIII \|AY228628** | A | G | . | . | A | T | . | A | . | A | . | A | T | A | G | . | A | . | C | . | . | A | . | C | T | . | G | . | T | C |  |  |  |  |  |  |  |  |  |
| **BIV \|L02116** | A | G | . | . | A | T | . | A | . | A | . | A | T | A | G | . | A | . | C | . | . | A | . | C | T | . | G | . | T | C |  |  |  |  |  |  |  |  |  |
| **C \|AY228641** | A | G | . | A | G | . | T | C | G | T | G | C | . | . | . | . | A | G | T | C | . | . | C | C | . | . | . | . | . | C |  |  |  |  |  |  |  |  |  |
| **D \|DQ246216** | A | T | C | . | . | T | . | C | A | . | . | A | . | T | . | . | A | . | C | . | . | T | C | A | T | A | T | . | T | . |  |  |  |  |  |  |  |  |  |
| **E \|AY655705** | . | . | A | . | . | . | G | A | . | T | C | . | . | A | . | A | A | . | A | C | . | T | . | . | . | . | . | A | . | . |  |  |  |  |  |  |  |  |  |
| **F \|AF069558** | . | . | . | . | . | T | . | . | . | G | . | . | . | . | . | . | . | G | A | . | . | T | . | C | . | . | . | - | - | - |  |  |  |  |  |  |  |  |  |
| **G \|EU781013** | A | A | . | . | . | . | T | . | . | . | G | A | . | . | . | . | A | . | T | C | A | . | . | C | . | A | T | . | . | C |  |  |  |  |  |  |  |  |  |

All the SNPs in the different genetic assemblages are displayed within *tpi* loci.
